# Acute Atherosis Lesions at the Fetal-Maternal Border: Current Knowledge and Implications for Maternal Cardiovascular Health

**DOI:** 10.3389/fimmu.2021.791606

**Published:** 2021-12-14

**Authors:** Daniel Pitz Jacobsen, Heidi Elisabeth Fjeldstad, Guro Mørk Johnsen, Ingrid Knutsdotter Fosheim, Kjartan Moe, Patji Alnæs-Katjavivi, Ralf Dechend, Meryam Sugulle, Anne Cathrine Staff

**Affiliations:** ^1^ Division of Obstetrics and Gynaecology, Oslo University Hospital, Oslo, Norway; ^2^ Institute for Clinical Medicine, Faculty of Medicine, University of Oslo, Oslo, Norway; ^3^ Department of Obstetrics and Gynaecology, Bærum Hospital, Vestre Viken HF, Bærum, Norway; ^4^ Experimental and Clinical Research Center, A Cooperation of Charité-Universitätsmedizin Berlin and Max-Delbruck Center for Molecular Medicine, Berlin, Germany; ^5^ Department of Cardiology and Nephrology, HELIOS-Klinikum, Berlin, Germany

**Keywords:** acute atherosis, inflammation, decidua basalis, preeclampsia, tolerance, cardiovascular disease, placenta, microchimerism

## Abstract

Decidua basalis, the endometrium of pregnancy, is an important interface between maternal and fetal tissues, made up of both maternal and fetal cells. Acute atherosis is a uteroplacental spiral artery lesion. These patchy arterial wall lesions containing foam cells are predominantly found in the decidua basalis, at the tips of the maternal arteries, where they feed into the placental intervillous space. Acute atherosis is prevalent in preeclampsia and other obstetric syndromes such as fetal growth restriction. Causal factors and effects of acute atherosis remain uncertain. This is in part because decidua basalis is challenging to sample systematically and in large amounts following delivery. We summarize our decidua basalis vacuum suction method, which facilitates tissue-based studies of acute atherosis. We also describe our evidence-based research definition of acute atherosis. Here, we comprehensively review the existing literature on acute atherosis, its underlying mechanisms and possible short- and long-term effects. We propose that multiple pathways leading to decidual vascular inflammation may promote acute atherosis formation, with or without poor spiral artery remodeling and/or preeclampsia. These include maternal alloreactivity, ischemia-reperfusion injury, preexisting systemic inflammation, and microbial infection. The concept of acute atherosis as an inflammatory lesion is not novel. The lesions themselves have an inflammatory phenotype and resemble other arterial lesions of more extensively studied etiology. We discuss findings of concurrently dysregulated proteins involved in immune regulation and cardiovascular function in women with acute atherosis. We also propose a novel hypothesis linking cellular fetal microchimerism, which is prevalent in women with preeclampsia, with acute atherosis in pregnancy and future cardiovascular and neurovascular disease. Finally, women with a history of preeclampsia have an increased risk of premature cardiovascular disease. We review whether presence of acute atherosis may identify women at especially high risk for premature cardiovascular disease.

## Introduction

Arterial lesions specific to the spiral arteries at the fetal-maternal border were first reported in 1945 (1). These lesions were later termed acute atherosis, described as lipid-laden foam cells within the intima, surrounded by fibrinoid necrosis and perivascular immune cell infiltrate (2, 3). Acute atherosis is associated with lower birthweight (4) and lower placental weight (5), and some studies show that acute atherosis may be correlated with an antiangiogenic profile (6, 7), all three of which are indicators of placental dysfunction. Moreover, the well documented high concomitance of acute atherosis and preeclampsia and other obstetric syndromes suggests shared underlying mechanisms (8–25).

Causal factors and effects of acute atherosis during pregnancy, as well as the long-term effects on maternal cardiovascular health, remain uncertain. There are several constraints on studying the acute atherosis lesions histologically. Systematic sampling of the decidua in large amounts following delivery is quite difficult, and a uniform, evidence-based research definition of acute atherosis lesions is historically lacking. As discussed below, both issues have been addressed by us (22, 26). However, even when these constraints are overcome, only a subset of the decidua can realistically be evaluated in any morphological tissue study, thus one can only with certainty determine the presence of acute atherosis, and never the definitive absence.

### Acute Atherosis Sampling Methodology

The ideal method for studying the impact of decidual acute atherosis on placental function requires specimens of a challenging nature; there are today only some very rare hysterectomy specimens of severely preeclamptic women with the placenta still *in situ* (27). In one such published case report, acute atherosis of spiral arteries with severe narrowing of the vascular lumen were associated with substantial infarcted areas in the overlying parts of the placenta (28). Moreover, in this case the lesions could be traced as deeply as the inner myometrium, implying that the severity of the placental defects may be related to the depth of the lesions. It is also noteworthy in this study that remodeling of the placental bed spiral arteries, including the myometrial segments, was absolutely normal in a few invaded spiral arteries at the very center of the placental bed. This suggests that non-invaded, more laterally situated vessels run a higher risk of developing acute atherosis, again highlighting the need for uniform sampling of relevant tissues for the study of acute atherosis, preferably of the whole placental bed.

Several methods have been employed for sampling decidua basalis for research purposes. These include placental bed biopsies (29), biopsies from the basal plate of delivered placentas (30), and our method of vacuum suctioning the placental bed (31). Of these sampling methods, placental bed punch or knife biopsy is the most invasive, but has the advantage of providing myometrium, which is needed if the goal is to study spiral artery remodeling or other features of this tissue (32). Biopsies from delivered placentas is the least invasive sampling method, and will yield moderate samples of decidua basalis tissue. However, if the goal is to study decidua basalis alone, our vacuum suction technique is the superior method (32–34).

The vacuum suction technique is performed during caeserean section, after delivery of the placenta, by applying vacuum suction to the uterine wall. This method has the advantages of an unbiased sampling and a large tissue yield. It is also time efficient and without danger to maternal health (34). One drawback is that tissue orientation is lost due to suctioning. Still, the vacuum suction method provides tissue applicable for acute atherosis research. We showed that higher rates of acute atherosis detection was achieved using vacuum suction samples, as compared to routinely sampled basal surface placental tissue and fetal membrane roll biopsies from the same pregnancies (33). The rate of decidual acute atherosis is thus likely underestimated in most studies, and we recommend using the vacuum suction technique if the goal is to study the lesions independent of tissue orientation. Our large Oslo Pregnancy Biobank, consisting of decidual tissue collected during elective cesarean section, along with placental tissue biopsies, fetal (umbilical artery as well as umbilical vein) and maternal blood samples, amniotic fluid, and maternal muscle and fat tissue biopsies, has enabled multiple studies comparing the presence of decidual acute atherosis and dysregulated features of other anatomical compartments (20–26, 31, 33, 35–41).

### Nonuniformity in Acute Atherosis Definitions

**Figure 1:**
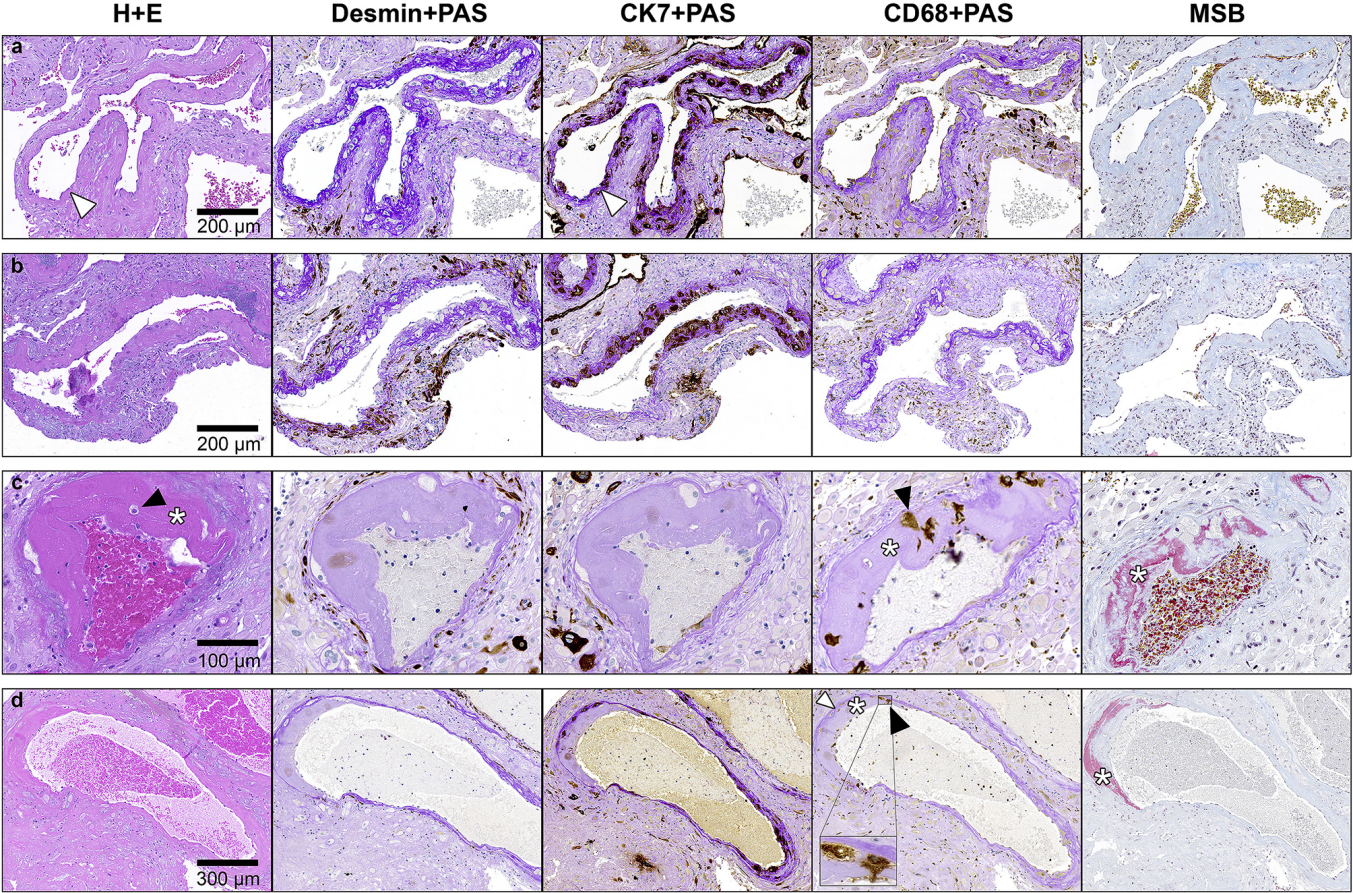
Staining of serial FFPE sections of decidua basalis tissue to identify spiral arteries. Slides are stained with (from left to right) Hematoxylin + Eosin (H + E), desmin and Periodic acid–Schiff (PAS), cytokeratin 7 (CK7) and PAS, CD68 + PAS and Martius Scarlet Blue (MSB). Representative images of **(A)** spiral artery from a normotensive control with complete physiological transformation, characterized by presence of CK7-positive trophoblasts and intramural fibrinoid (bright purple upon PAS staining, white arrowhead) in the vessel wall, and complete absence of intramural smooth muscle cells (no desmin stain). **(B)** Spiral artery from a preeclampsia patient with partial physiological transformation characterized by intramural fibrinoid, trophoblasts and areas with traces of mural smooth muscle cells (desmin-positive). **(C)** Spiral artery with acute atherosis from same sample as in **(B)**, lacking bright purple fibrinoid and CK7-positive trophoblasts in the vessel wall. Traces of intramural smooth muscle cells (desmin positive) are seen. Fibrinoid necrosis is visible as a grey-pink material in the vessel wall (asterisk), which stains red upon MSB staining (asterisk). Erythrocytes in the lumen of the AA artery stains red-brown upon MSB staining. Intramural CD68-positive foam cells are present (black arrowhead). **(D)** Spiral artery from a preeclampsia patient with almost complete physiological transformation – evident by the lack of desmin-positive smooth muscle cells and the presence of CK7-positive trophoblasts – yet acute atherosis lesion is present (asterisk; fibrinoid necrosis, black arrowhead; foam cells, white arrowhead; purple physiological fibrinoid). Inset; higher power inset of foam cells. Reprinted from Fosheim et al. (35), with permission from the journal *Placenta*.

Historically, a uniform definition of acute atherosis has been lacking. This may have led to discrepancies in reported rates of acute atherosis across pregnancy groups. In addition, differences in patient populations studied as well as in tissue collection and evaluation methodology (e.g. antibody selection) may have contributed. Moreover, clear definitions of perivascular infiltrate (PVI) and fibrinoid necrosis have been lacking. We set out to address these issues by attempting to establish an evidence-based research definition of acute atherosis (22). After examining 278 decidua basalis samples, we observed that perivascular leukocyte infiltrates and increased fibrinoid did not always correlate with adjacent foam cell lesions. Instead, we concluded that these are features of the decidual pathology of preeclampsia, while CD68^+^ foam cells are an essential aspect of acute atherosis (**Figure 1**). Thus, we proposed that acute atherosis should be diagnosed solely by the presence of foam cell lesions, defined as two or more intramural, adjacent, vacuolated CD68^+^ cells. Nonetheless, throughout this review, we will include studies with other acute atherosis diagnosis criteria as well.

## Acute Atherosis Etiology

Hertig, who first described acute atherosis, proposed that vessel damage followed by lipophage infiltration is what initiates acute atherosis lesion development (1). Endothelial injury has long been suspected as integral to decidual lesion development by others as well (42). However, the lack of an association between acute atherosis and the severity and duration of hypertension, or antihypertensive treatment, implies that hemodynamic forces alone are not adequate for lesion development (43). Similar to the heterogeneity of preeclampsia, acute atherosis is likely a multifactorial pathology with several pathways leading to an adverse uterovascular phenotype endpoint. We now have access to 75 years of research into the nature of the histological lesions known as acute atherosis, but have not finished elucidating the complexity of its etiological and mechanistic molecular constituents.

One such potential constituent, however, may be endothelin-1 (ET-1) (44). ET-1 is a highly potent vasoconstrictor (45). It is upregulated by mechanical stretch (46) and hypoxia (47), and plasma ET-1 is elevated in preeclampsia and gestational hypertension (48, 49). It exerts its effects by binding G-protein coupled receptors on vascular smooth muscle cells and endothelial cells (50), a byproduct of which may be intracellular lipid accumulation (51, 52). Thus, ET-1 may be a common trigger for lipid accumulation within endothelial cells in acute atherosis and in hepatocytes in the associated rare disease, acute fatty liver of pregnancy (44).

Another vasoconstrictor of interest is angiotensin II, which may play a role in the pathogenesis of atherosclerosis (53). We have postulated a role in acute atherosis for activating antibodies against the angiotensin II type 1 receptor (AA-AT_1_), after demonstrating a clear association between AA-AT_1_ and preeclampsia (54–56). Based on our cesarean delivery population, we were unable to demonstrate any association between AA-AT_1_ and acute atherosis (21). However, angiotensin II is known to work synergistically with ET-1 (57), and studying both of these vasoconstrictor systems simultaneously would shed more light on the possible involvement of G-protein cascades in acute atherosis development.

The regulator of G protein signaling 2 (RGS2) likely has implications for ET-1 and AA-AT_1_ signaling (58). Interestingly, we have observed an association between acute atherosis and a genotype associated with lower RGS2 expression (38). If G-protein cascades indeed cause intracellular lipid accumulation, as proposed by Coffey (44), we would expect early acute atherosis lesions to contain lipid-laden endothelial or vascular muscle cells. Accordingly, arterial lesions containing vacuolated endothelial cells and myofibroblasts have been observed in first-trimester curetted endometrium samples from therapeutic and spontaneous abortions (59, 60). A higher incidence of such lesions was observed in primigravida as compared to multigravida (60). Primigravidity is associated with an increased risk of preeclampsia (36, 61), often considered due to excessive inflammation, although evidence is lacking (62). Whether these lesions are precursors to full-blown acute atherosis, and whether lipid accumulation within endothelial cells and myofibroblasts is indeed the insult that catalyzes intramural immune cell infiltration, remains to be investigated.

Acute atherosis shares morphological features with *early* atherosclerotic lesions, which is recognized as an inflammatory disease of the arterial walls (63). Both lesions present with increased numbers of intimal macrophages, lipid-laden foam cells, lipoprotein(a) throughout the vessel walls and extracellular droplets of lipid as well as similar expression of intracellular lipid-handling proteins (41, 64–67). Moreover, both acute atherosis and atherosclerosis are associated with preeclampsia and other states of systemic inflammation (68, 69).

However, there are several differences between acute atherosis and atherosclerosis. Firstly, vessel caliber differs enormously. Atherosclerosis is found in major arteries with a thick intimal layer, and the vessels are supplied with oxygen and nutrients from the vasa vasorum (70). Notably, the vasa vasorum may be instrumental as a source of lipids in the development of atherosclerosis (71–73). Spiral arteries are much smaller and do not have an external blood supply. Preeclampsia is associated with elevated lipid content in decidua basalis tissue, which may act as a source of lipid compounds for lesion development (26). Moreover, acute atherosis is not associated with *plasma* lipid contents, further indicating a local rather than a systemic excess of lipids (23). Secondly, research into the molecular composition of acute atherosis versus atherosclerosis reveals several dissimilarities. For instance, we have observed no LOX-1 positive endothelial cells or foam cells within the lesions of spiral arteries (41), while this lipid scavenger receptor is a key contributor to atherosclerotic development (74). Finally, endothelial activation is important in atherosclerosis (75), whereas evidence is conflicting with regards to endothelial status in acute atherosis. Although one study reported endothelial and interstitial extravillous trophoblast ICAM-1 expression in placentas with acute atherosis (76), we were unable to detect any ICAM-1 expression within the acute atherosis lesions (35). Moreover, the endothelial lining of the artery wall is often destroyed in acute atherosis and there is evidence of leakage of fibrin-like material from the circulation into the vessel walls (35, 42, 77). Accordingly, in a study of women with preeclampsia, we demonstrated elevated levels of thrombomodulin – a marker of endothelial dysfunction (78) or damage (79) – in those who had concomitant acute atherosis (39).

Acute atherosis is not found outside of the uterus (80). The lesions are focal and patchy, mainly localized downstream in the circulation, at the tips of the decidua basalis spiral arteries. The major remodeling problems occur upstream in the myometrium (67, 80). Yet, there is a link between acute atherosis formation and poor remodeling, as lesions are commonly found downstream of inadequately remodeled spiral arteries (77). The fully remodeled decidual segment of the spiral artery may be considered as being somewhat “naked” and is then likely more at risk for attacks both from the inside (by luminal, circulating factors) as well as from the outside (by components in the surrounding decidual tissue) in addition to being especially exposed to ischemia-reperfusion injury due to turbulent blood flow. Specifically, we postulate that these areas are especially exposed to local inflammatory signaling molecules. This may be compounded by their unique local environment close to the semi-allogenic fetal cells and the resulting inflammatory changes, potentially explaining why this uteroplacental location seems to be a prerequisite for the development of these particular atherosis lesions.

Inflammation does indeed appear to be clearly linked to acute atherosis development. Spiral arteries affected by acute atherosis contain huge deposits of IgM, as well as smaller amounts of IgG and IgA within the arterial wall. In addition to immunoglobulins, complement component 3 (C3) is often observed within acute atherosis lesions (81–86). In addition, early immunohistochemistry studies of the leukocyte infiltrate demonstrated T lymphocytes in acute atherosis (87). We later expanded on these findings, demonstrating increased concentrations of CD3^+^, CD8^+^ and CD3^+^CD8^-^ intramural T cells in the walls of spiral arteries with acute atherosis compared to arteries from samples without acute atherosis (37). Higher numbers of CD3^+^ and CD3+CD8^-^ T cells were also observed in the surrounding perivascular space. Furthermore, a study by Gill and colleagues conducting flow cytometry of basal plate samples demonstrated higher numbers of M1-macrophages in acute atherosis, displaying a pro-inflammatory phenotype (88). Fluorescence staining revealed M1-macrophage localization within vessel walls of spiral arteries affected by acute atherosis. Interestingly, a recent study from India showed that the incidence of acute atherosis – after exclusion of placental associated syndromes like fetal growth restriction, pregnancy hypertension and diabetes – was significantly higher in asymptomatic or mildly symptomatic SARS-CoV-2 positive pregnant women as compared to SARS-CoV-2 negative pregnant women (89). Finally, another support of acute atherosis representing an inflammatory lesion is its resemblance to systemic vasculitis, a general term applied to inflammation of vessel walls that progresses to fibrinoid necrosis (90, 91).

## Many Roads to Decidual Inflammation

**Figure 2:**
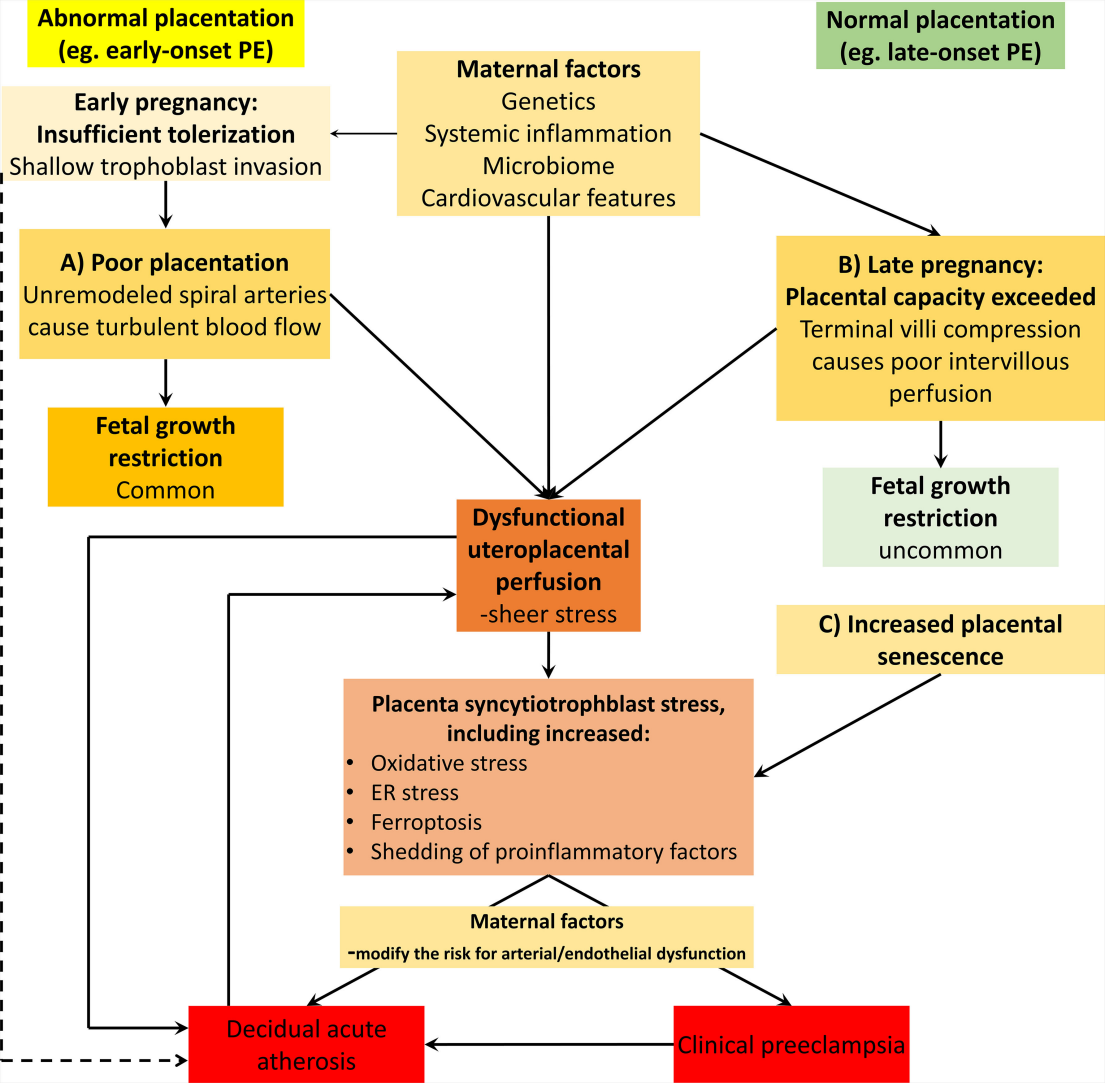
Acute atherosis presence across the several forms of placental syndrome forms. Acute atherosis has been found in several forms of placental syndromes, many of these associated with spiral artery remodeling deficiencies, including early-onset preeclampsia (PE) (illustrated on the left side of the Figure). This figure also illustrates our concepts of how acute atherosis may be present also in late forms of preeclampsia (illustrated on the right side of the Figure), or indeed in any other forms of placental dysfunction without spiral artery remodeling defects. Preplacentation factors impacts the early and ensuing placentation processes, and includes both fetal-maternal tolerization processes as well as endometrial health (92). In this model, acute atherosis is seen as a consequence of any form of placental dysfunction and its underlying mechanisms (69). The triggers include both the uteroplacental malperfusion pathway secondary to spiral artery remodeling problems (as in early-onset preeclampsia) as well as the late-onset preeclampsia forms with other causes of placental malperfusion and syncytiotrophoblast stress (92, 93). Furthermore, this model also proposes that acute atherosis itself may represent a risk factor for placental dysfunction and preeclampsia. This is in line with acute atherosis developing very early in women with excessive vascular inflammation, such as in systemic lupus erythematosus, who also have a high risk for developing early-onset preeclampsia, severe fetal growth restriction and intrauterine death, all severe clinical aspects of placental dysfunction. The line from the tolerization box to acute atherosis illustrate how HLA-C/KIR interactions and alterations in level of immune-dampening molecules such as sHLA-G could contribute to lesion promotion at the maternal-fetal interface, as discussed in this review. The maternal factors promoting the clinical forms of preeclampsia (e.g. early- and late-onset) as well as atherosis development include cardiovascular, inflammatory and metabolic factors. Also, any form of clinical preeclampsia, with excessive circulation of proinflammatory factors and hypertension may add to the risk for atherosis development, although hypertension in itself is not a mandatory requirement.

We hypothesize that several mechanisms may trigger acute atherosis. Our hypothesis places inflammation at the center of lesion and as the final common pathway converged upon by these different triggers (69). We believe these mechanisms may act individually or in concert to produce acute atherosis, as illustrated in **Figure 2**.

### Maternal Alloreactivity

Human pregnancy is a dynamic balancing act for the maternal immune system. The fetal allograft must peacefully coexist with the maternal immune and cardiovascular systems, whilst the mother and fetus simultaneously remain protected against microbial infections. Under any other circumstances, immune cells would quickly target genetically foreign tissue. However, a cascade of immune-modulating molecules acting throughout pregnancy enable the conceptus to evade rejection until parturition. The innate immune system is strengthened, while adaptive immunity is weakened (94). Serial blood samples collected at different time points during pregnancy have revealed precise timing of particular immunological changes (95). Aghaeepour and colleagues suggest deviations from this “immune clock of human pregnancy” could indicate pregnancy-related pathologies.

The fetus inherits half of its genetic material from the mother, and the other half from the father. Given the extremely high variability in major histocompatibility complex (MHC) genetics, it is unlikely that the paternally inherited fetal MHC alleles are identical to the maternally inherited fetal MHC alleles. Fetal trophoblasts circumvent this obstacle and avoid rejection by local maternal immune cells by not expressing most classical MHC class I or class II surface molecules (96). They do, however, express human leukocyte antigen (HLA) C and the non-classical MHC molecules HLA-G, HLA-E and HLA-F (96–98). HLA-C and HLA-G attenuate immune activation by binding to killer immunoglobulin like receptors (KIR), which are abundantly expressed on maternally derived immune cells (99, 100). KIR activation prevents cytotoxicity by alloreactive T-cells (101), and may induce apoptosis of activated T-cells and NK cells (102, 103). Invading extravillous trophoblasts rely on these KIR/HLA-interactions to avoid immune cell attacks. In fact, there is a positive correlation between the amount of surface HLA-G expression and the depth of trophoblast invasion into the decidua (104).

Several factors may influence these tolerogenic pathways. As HLA-C is the only polymorphic histocompatibility antigen expressed by fetal cells at the fetal-maternal interface, paternal HLA-C genotype is particularly important. In fact, HLA-C mismatched pregnancies are characterized by a higher percentage of activated maternal T-cells (105). Moreover, the combination of fetal HLA-C and maternal killer immunoglobulin like receptor (KIR) genotypes may greatly predispose pregnancies to preeclampsia (106). We have expanded on this finding by showing that the combination of fetal HLA-C2 with the maternal KIR-B haplotype was significantly associated with acute atherosis in preeclampsia (25).

Similarly, inadequate induction of tolerance by HLA-G is detrimental to pregnancy health (107). Membrane bound HLA-G expression is lower on trophoblasts from preeclamptic placentas (108, 109). Circulating soluble HLA-G (sHLA-G) is also lower in preeclampsia throughout all trimesters, compared to pregnancies that remain normotensive (110). We have shown that maternal sHLA-G inversely correlates with the level of placental dysfunction, the latter evaluated by maternal levels of the antiangiogenic factor sFlt-1, or by the sFlt-1/PlGF ratio (36), and that fetal polymorphisms in the 3’UTR region of HLA-G are associated with presence of acute atherosis in preeclampsia (24). Our hypothesis is that these polymorphisms lead to altered HLA-G expression in the decidua basalis, affecting local fetal-maternal immune tolerance and in turn promoting development of acute atherosis. Failure to establish fetal-maternal tolerance may also influence trophoblast invasion into the decidua. These extravillous trophoblasts are involved in the plugging and remodeling of uteroplacental spiral arteries in early pregnancy (80).

### Ischemia-Reperfusion Injury

The first 10 weeks of pregnancy, the spiral arteries extending from the placenta to the endometrial surface of the decidua are effectively plugged, and the fetus exists in a state of physiological hypoxia (111, 112). At the end of the first trimester, the maternal vessels of the decidua open up and the placenta is submerged in maternal blood (111). This marks a dramatic shift in the fetoplacental exposure to the maternal cardiovascular and system.

Throughout pregnancy, uteroplacental blood flow increases, reaching up to 750 ml/minute at term, about 25% of maternal cardiac output (113). At the same time, the approximate 5-fold increase in diameter of the terminal coils of fully remodeled spiral arteries dramatically slows down the speed of the blood entering the intervillous space (111). Failure of proper spiral artery remodeling results in downstream placental malperfusion. The retention of smooth muscle within the arterial wall likely causes ischemia-reperfusion injury (114), and not impaired flow volume nor uteroplacental hypoperfusion. In addition, we argue that placental malperfusion may not be exclusively secondary to failure of spiral artery remodeling. Failed remodeling may be considered an “external” cause of placental malperfusion and is typically seen in early-onset preeclampsia. Late-onset preeclampsia affects a greater rate of women than the early-onset form, and spiral artery remodeling is rarely affected. In this setting, malperfusion may be caused by two “internal” pathways. In the one pathway, placental senescence causes a syncytiotrophoblast stress response as the pregnancy progresses towards term and thereafter. The other pathway occurs with particularly large placentas, such as in multiple gestations, in which compression leads to placental congestion and thereby malperfusion (93, 115). The ensuing disturbances in calcium-homeostasis may cause endoplasmic reticulum stress and initiate the unfolded protein response, ultimately leading to cell death (116, 117). Moreover, intracellular buildup of reactive oxygen species induces upregulation and secretion of the proinflammatory cytokines TNF and IL-1b (118, 119). ER stress and oxidative stress in placental tissues are both features of preeclampsia (31, 117, 120), and may also play a role in the strongly associated acute atherosis lesion development.

High pressure and turbulent blood flow may also damage the endothelial lining of the terminal coils of spiral arteries, as well as syncytiotrophoblasts coating placental villi. Analysis of hemodynamic forces on vascular endothelial cells has shown that disturbed blood flow and continuous low grade shear stress acting on the arterial wall may promote atherogenesis (121). This is in line with the increased incidence of atherosclerotic lesions at arterial branch points or sections with high curvature (122, 123). Endothelial cells possess surface molecules capable of detecting shear stress and inducing gene transcription through the Ras-MAP kinase signaling pathway (124). Among several changes in gene expression is a transient upregulation of the monocyte chemotactic protein 1 (MCP-1) (125). Overexpression of MCP-1 attracts macrophages and may induce infiltration into vessel walls (126). Interestingly, endothelial MCP-1 is upregulated in preeclampsia (127, 128), possibly due to atherogenic blood flow, and could possibly be involved in CD68^+^ cell recruitment to the sites of lesion development (129, 130).

Ferroptosis is a recently discovered mode of iron-dependent cell death (131, 132). Lipoxygenases and other enzymes may induce ferroptosis in a controlled manner (133). In addition, and more relevant to the topic at hand, ferroptosis may occur due to iron dysregulation and free-radical chain reactions, leading to hydroxyl and peroxyl radicals (133). Recently, ferroptosis has gained attention as a possible target against ischemia-reperfusion injury (134–136). Thus, ferroptosis may play a role in early-onset preeclampsia following incomplete spiral artery remodeling. Interestingly, huge amounts of iron have been observed in atherosclerotic lesions (137), and lipid peroxidation is known to play a significant role in atherogenesis (138). Moreover, animal experiments have shown alleviation of atherosclerosis through inhibition of ferroptosis (139). However, the role of iron-dependent cell death due to ischemia-reperfusion in acute atherosis development remains to be investigated.

### Preexisting Systemic Inflammation

Systemic inflammation is associated with many chronic diseases, such as obesity, diabetes mellitus and cardiovascular disease, as reviewed in (140–142). Considering that even normal pregnancy is associated with elevated systemic inflammation (143), one would expect heightened baseline inflammation to be associated with higher rates of obstetric complications linked to the maternal immune system. This is indeed what is observed. Obesity, diabetes mellitus and high blood pressure are all substantial risk factors for miscarriage (144–146), preeclampsia (92, 147) and fetal neurodevelopmental disorders (148–150). In line with the effects of other chronic inflammatory conditions on pregnancy, pregnant women with autoimmune disease experience higher rates of hypertensive disorders of pregnancy, intrauterine growth restriction, preterm delivery and autism spectrum disorder in their offspring (151–154). Interestingly, autoimmune disorders are also commonly observed in association with acute atherosis (155, 156). In fact, lesions have been observed as early as the first trimester in women with systemic lupus erythematosus (157).

### Microbial Infection

Many tissues first thought to be sterile have been shown to harbor dormant bacteria. This includes blood (158, 159), seminal fluid (160) and possibly the placenta (161) – although this latter claim is disputed (162). The source of these bacteria may be the gut (163), the oral cavity (164) or the urinary tract (165). The iron dysregulation and dormant microbes hypothesis proposes these bacteria may be resuscitated from dormancy by free iron and manifest a diverse range of chronic inflammatory diseases previously thought to not possess infectious properties such as preeclampsia and atherosclerotic disease (166). Viable bacteria release lipopolysaccharide (LPS) or lipoteichoic acid (LTA). This initiates a cascade of immune responses, including a dramatic upregulation of many circulating cytokines and other acute phase signaling molecules like serum amyloid A1 (SAA1) and C-reactive protein (167, 168). As extensively reviewed by Kell and Kenny, there are several lines of evidence pointing towards microbial contribution in the development of preeclampsia (169). There is high co-occurrence between bacterial infections and preeclampsia. Examples include *Chlamydia pneumoniae* (170, 171) and *Helicobacter pylori* (172). Several biomarkers associated with preeclampsia have also been linked to microbial infections, including sFlt-1/PlGF ratio (173, 174) and SAA1 (167). In support of the concept that preeclampsia development has a microbial component, is the virtual absence of preeclampsia in pregnancies with *Toxoplasma gondii* infection treated with anti-microbial medication (spiramycin) (175). Kell and others have also argued for the existence of a microbial component in atherosclerosis with quite compelling evidence (159, 166, 176). Patients with chronic bacterial infections are substantially more at risk for atherosclerosis (177, 178), and LPS is regularly used to generate animal models of the disease (179, 180). Moreover, atherosclerotic plaques contain bacterial DNA (181, 182) and elevated levels of iron (180), adding credence to the iron dysregulation and dormant microbes hypothesis. The similarities to atherosclerosis have led others to speculate that an infectious trigger underlies the development of acute atherosis as well (183, 184). However, this matter remains unsettled.

## A New Hypothesis Linking Cellular Fetal Microchimerism With Acute Atherosis in Pregnancy and Future Cardiovascular and Neurovascular Disease

Cellular fetal microchimerism (cFMC) arises when cells of fetal origin are transferred to maternal blood and tissues during pregnancy (185). These cells are known to possess stem cell-like properties, capable of differentiating into endothelial cells, smooth muscle cells and even leukocytes (186, 187). During pregnancy, a lot of fetal material leaks into maternal circulation. While cell free fetal DNA and other debris is rapidly cleared following delivery (188) and likely completely absent from maternal systems postpartum, cFMC may persist for decades. In fact, fetal cells have been observed in maternal circulation up to 27 years postpartum, indicating that these cells may inhabit maternal systems throughout life (189). Restorative as well as detrimental effects have been attributed to cFMC, possibly tied to fetal-maternal histocompatibility (185). Of particular interest in the context of this review is the apparent detrimental effect of cFMC on autoimmunity (190, 191). The trigger has been proposed to be a maternal alloimmune response towards fetal cells expressing foreign HLA surface peptides (191). By far the majority of patients diagnosed with autoimmune disorders are women (192), which could partly be due to the acquisition of fetal cells during pregnancy.

In comparison to healthy pregnancies, circulating fetal microchimeric cells are more prevalent in pregnancies complicated by preeclampsia (193, 194) or severe fetal growth restriction combined with impaired placental perfusion (195). If this is due to increased cell transfer, reduced clearance or reduced migration from maternal blood into maternal tissues is unclear (185, 196). We hypothesize that when the placenta is dysfunctional, fetal cells leak more freely across into maternal blood and subsequently other tissues. These cells may then inhabit maternal vessels, in the presence or absence of endothelial damage, and induce a maternal anti-fetal immune response towards the vascular endothelium. This could explain the association between male cFMC and an increased cardiovascular mortality hazard ratio (197). However, this last observation was based on a total of only 5 cardiovascular deaths. A recent study on a much larger cohort found that male-origin microchimerism was associated with *reduced* risk of ischemic heart disease as well as no association between microchimerism and ischemic stroke (198).

Microchimerism is not unique to pregnancy. Low levels of donor cells may be acquired following solid organ transplantation (199). The presence of such microchimerism has been linked to graft acceptance (200) as well as graft rejection (201). As with autoimmune diseases and cFMC, the effect of donor microchimerism may depend on how well the host tolerates the graft. Interestingly, vascular lesions have been observed in arteries surrounding transplanted organs following kidney transplant rejection (202), after liver transplantation (203) and after heart transplantation (204). These lesions are characterized by large amounts of lipids, IgM and C3 (205), and thus bear striking resemblance to acute atherosis.

Our novel hypothesis states that placental dysfunction leads to augmented cFMC. If persistent in the circulation or alternatively engrafted in maternal endothelium, these semi-allogenic cells could cause further inflammation, particularly in vessel walls, and initiate development of inflammatory arterial lesions such as acute atherosis. As cFMC persist decades after pregnancy, there may be a role for these cells in the pathogenesis of chronic cardiovascular and neurovascular disease in the long term as well. We believe this hypothesis merits further testing in translational clinical studies.

## Clinical Translation

### During Pregnancy

Acute atherosis is associated with arterial thrombosis, placental infarction and perinatal death (22, 28, 30, 206, 207). This has led researchers to propose aspirin as a possible treatment for acute atherosis (208). Aspirin is an effective prophylactic treatment against thrombosis (209). Outside of pregnancy, aspirin is widely used for primary and secondary prevention of atherosclerotic CVD (210). Daily low-dose aspirin started during the first trimester has shown a substantially reduced risk of preeclampsia in women at high risk (211–213). Whether this effect is related to a reduced tendency of blood clot formation, and how this may relate to acute atherosis, remains to be investigated.

Acute atherosis lesions may also effectively reduce the diameter of uteroplacental spiral arteries, causing aberrant blood flow (69, 111). The evidence of this latter claim is, however, conflicting. Supporting this, an early study of only 6 cases showed a trend between acute atherosis and high uterine artery pulsatility index, which could reflect obstructed spiral artery blood flow (214). Furthermore, another research group showed that acute atherosis is associated with a higher incidence of placental lesions characteristic of maternal vascular hypoperfusion (215). Placental lesions are also associated with abnormal uterine velocimetry measurements among women with intrauterine fetal growth restriction (216). In contrast to these findings, one study found no association between uterine artery pulsatility index and acute atherosis (217). The reasons for these discrepant findings may be that the spiral arteries are not visualized directly by Doppler studies, but rather indirectly by studying the blood flow of the larger uterine arteries (214, 216–218). Uterine and spiral arteries differ much in structure, size and function. Uterine artery ultrasonography has historically been viewed as a reflection of spiral artery remodeling (219), but recent studies indicate that the radial arteries, as well as the maternal vascular system, may have a larger impact on uterine artery waveforms than the spiral arteries (218). Doppler ultrasonography is hence unlikely a reliable tool for diagnosing uteroplacental acute atherosis.

### Postpartum: Targeting the Women at Highest Risk of Premature Cardiovascular Disease?

We have put forth the hypothesis that women with concomitant preeclampsia and acute atherosis are at especially high risk for developing atherosclerosis and premature cardiovascular disease (130). If pregnancy is a physiological stress test (220, 221), then preeclampsia is an unmasking of compromised maternal cardiovascular health. Time and time again preeclampsia has been linked to future maternal cardiovascular disease in one form or another (222–230). The severity of preeclampsia also correlates with ischemic heart disease incidence rate (231). Moreover, fetal manifestations of placental dysfunction, such as intrauterine growth restriction, further add to maternal risk of cardiovascular disease (232). Abnormal placentation also associates with infertility or subfertility, both of which are associated with poor long-term cardiovascular health (233, 234). As described above, acute atherosis may disturb placental perfusion (69, 111), and is associated with low birth weight (4) and low placental weight (5). Acute atherosis may thus play a role in producing and/or exacerbating maternal and fetal symptoms of placental dysfunction, associated with high cardiovascular disease risk.

There are striking similarities between acute atherosis and atherosclerosis, indicative of shared pathophysiology. We know that preeclampsia is associated with a substantial atherosclerotic load (235). Compared to normotensive women and women with gestational hypertension, women with preeclampsia have higher carotid intima-media thickness (CIMT) (236, 237) – increasingly used as a surrogate marker for preclinical atherosclerosis (238). In some studies, these differences remained evident up to 18 months postpartum (236, 237). However, other studies with longer follow-up did not corroborate these findings (239, 240). Notably, one study unexpectedly reported *thinner* CIMT 7 Years postpartum in women with preeclampsia compared to controls (241). Instead, they found increased intima thickness as well as intima-media ratio in cases versus control. The authors suggest that these measures are preferable to the conventional CIMT for assessing cardiovascular disease risk in women with a history of preeclampsia. As lesions of the placental bed manifest during a shorter time span compared to atherosclerosis (possibly due to the proximity to the foreign fetus and the excessive inflammation of pregnancy), acute atherosis may possibly be used as an indicator of women with excessive atherosclerotic load (130).

Several studies have examined the link between decidual lesions and subsequent cardiovascular health. Two retrospective cohort studies by the same research group have revealed long-term cardiovascular consequences of decidual vasculopathy (242, 243). Decidual vasculopathy was in these studies defined by vascular fibrinoid necrosis and lipid-filled foam cells in the vascular wall, thus sharing some of the features traditionally used for acute atherosis (244). The first study examined cardiovascular parameters 2-74 months postpartum in women whose index pregnancies were complicated by preeclampsia. Women with concomitant decidual vasculopathy and preeclampsia had higher diastolic blood pressure, lower left ventricular stroke volume and higher total peripheral vascular resistance as compared to women with only preeclampsia (243). Decidual vasculopathy did, however, not correlate with circulating lipids or thrombophilia postpartum. The second study demonstrated a higher prevalence of chronic hypertension several years postpartum in women who had concomitant decidual vasculopathy and preeclampsia, compared to women who only had preeclampsia. These results remained significant after correcting for chronic hypertension before index pregnancy (242).

A small study comprising only 3 cases of acute atherosis showed higher levels of triglycerides and low-density lipoprotein in these women on the first day postpartum as compared to women without acute atherosis (245). Our group conducted a larger study where we also measured triglycerides and cholesterols (among other circulating biomarkers) the day of cesarean section. We observed no differences in circulating cardiovascular biomarkers between women with acute atherosis and women without. However, when restricting the analyses to women of advanced maternal age (age 36-44) we observed significantly elevated low-density lipoprotein and ApoB in women with acute atherosis (20). These studies highlight the potential use of acute atherosis in targeting women at particularly high risk of cardiovascular disease – a concept promoted by us and others previously (246, 247). Utilizing the more readily detected maternal vascular malperfusion lesions of the placenta has also been suggested (248), although some claim cardiovascular disease risk should be linked with atherosclerotic lesions of the uteroplacental artery instead of decidual basal artery or placental lesions (249).

## Future Research Opportunities

As outlined above, there is substantial evidence backing up the concept of acute atherosis as a pregnancy-specific inflammatory arterial lesion. However, many uncertainties regarding acute atherosis remain. Firstly, the risk factors and triggers that initiate lesion development have not been fully elucidated, though there seems to be a consensus among researchers that endothelial damage is part of it. Damage could stem from ischemia-reperfusion injury, infections or excessive activation of G-protein cascades, to name a few. Further research into the molecular constituents of acute atherosis – in particular early lesion stages – could shed some light on this issue. Secondly, there are many candidates for drivers of inflammation and lesion development following endothelial insult. Further knowledge of which pathways play a substantial role could guide the development of prophylactic treatments of obstetric syndromes tightly associated with acute atherosis. This includes our suggestion of testing whether the use of anti-atherogenic statins during severe preeclampsia or fetal growth restriction, such as in women with systemic lupus, may ameliorate acute atherosis, improve uteroplacental perfusion and enhance pregnancy outcome (69). Thirdly, the long-lasting implications of pregnancy affected by acute atherosis on maternal health need further research. There is a clear lack of studies with hard endpoints to show if acute atherosis, as we have proposed, can be used to identify women at substantial risk for premature cardiovascular disease and death.

## Author Contributions

DPJ wrote the review. HEF, GMJ, IKF, KM, PA-K, RD, MS, and ACS revised the manuscript and gave expert scientific input on its content. All authors contributed to the article and approved the submitted version.

## Funding

DPJ and HEF receive salaries for the MATCH (ref 2019012) and FETCH (ref. 2017007) studies funded by the South-Eastern Norway Regional Health Authority, as well as the BRIDGE (ref. 313568) study funded by the Research council of Norway.

## Conflict of Interest

Author RD was employed by company HELIOS-Klinikum GmbH.

The remaining authors declare that the research was conducted in the absence of any commercial or financial relationships that could be construed as a potential conflict of interest.The handling editor declared a past collaboration with one of the authors, AS.

## Publisher’s Note

All claims expressed in this article are solely those of the authors and do not necessarily represent those of their affiliated organizations, or those of the publisher, the editors and the reviewers. Any product that may be evaluated in this article, or claim that may be made by its manufacturer, is not guaranteed or endorsed by the publisher.

## References

[1] Hertig AT. Vascular Pathology in the Hypertensive Albuminuric Toxemias of Pregnancy. Clinics (1945) 4:602–14.

[2] Zeek PM Assali NS. Vascular Changes in the Decidua Associated With Eclamptogenic Toxemia of Pregnancy. Am J Clin Pathol (1950) 20(12):1099–109. doi: 10.1093/ajcp/20.12.1099 14783095

[3] Sexton LI Hertig AT Reid DE Kellogg FS Patterson WS. Premature Separation of the Normally Implanted Placenta; a Clinicopathological Study of 476 Cases. Am J Obstet Gynecol (1950) 59(1):13–24. doi: 10.1016/0002-9378(50)90335-8 15408710

[4] Frusca T Morassi L Pecorelli S Grigolato P Gastaldi A. Histological Features of Uteroplacental Vessels in Normal and Hypertensive Patients in Relation to Birthweight. Br J Obstet Gynaecol (1989) 96(7):835–9. doi: 10.1111/j.1471-0528.1989.tb03324.x 2765429

[5] Stark MW Clark L Craver RD. Histologic Differences in Placentas of Preeclamptic/Eclamptic Gestations by Birthweight, Placental Weight, and Time of Onset. Pediatr Dev Pathol (2014) 17(3):181–9. doi: 10.2350/13-09-1378-OA.1 24625285

[6] Korzeniewski SJ Romero R Chaiworapongsa T Chaemsaithong P Kim CJ Kim YM. Maternal Plasma Angiogenic Index-1 (Placental Growth Factor/Soluble Vascular Endothelial Growth Factor Receptor-1) is a Biomarker for the Burden of Placental Lesions Consistent With Uteroplacental Underperfusion: A Longitudinal Case-Cohort Study. Am J Obstet Gynecol (2016) 214(5):629 e1– e17. doi: 10.1016/j.ajog.2015.11.015 PMC5769706 26688491

[7] Baltajian K Hecht JL Wenger JB Salahuddin S Verlohren S Perschel FH. Placental Lesions of Vascular Insufficiency are Associated With Anti-Angiogenic State in Women With Preeclampsia. Hypertens Pregnancy (2014) 33(4):427–39. doi: 10.3109/10641955.2014.926914 25062083

[8] Ghidini A Salafia CM Pezzullo JC. Placental Vascular Lesions and Likelihood of Diagnosis of Preeclampsia. Obstet Gynecol (1997) 90(4 Pt 1):542–5. doi: 10.1016/S0029-7844(97)00360-8 9380313

[9] Brosens I Dixon HG Robertson WB. Fetal Growth Retardation and the Arteries of the Placental Bed. Br J Obstet Gynaecol (1977) 84(9):656–63. doi: 10.1111/j.1471-0528.1977.tb12676.x 911717

[10] De Wolf F Brosens I Renaer M. Fetal Growth Retardation and the Maternal Arterial Supply of the Human Placenta in the Absence of Sustained Hypertension. Br J Obstet Gynaecol (1980) 87(8):678–85. doi: 10.1111/j.1471-0528.1980.tb04601.x 7426529

[11] Althabe O Labarrere C Telenta M. Maternal Vascular Lesions in Placentae of Small-for-Gestational-Age Infants. Placenta (1985) 6(3):265–76. doi: 10.1016/S0143-4004(85)80056-4 4022955

[12] McFadyen IR Price AB Geirsson RT. The Relation of Birthweight to Histological Appearances in Vessels of the Placental Bed. Br J Obstet Gynaecol (1986) 93(5):476–81. doi: 10.1111/j.1471-0528.1986.tb08657.x 3707878

[13] Khong TY. Acute Atherosis in Pregnancies Complicated by Hypertension, Small-for-Gestational-Age Infants, and Diabetes Mellitus. Arch Pathol Lab Med (1991) 115(7):722–5. 2064534

[14] Pijnenborg R Anthony J Davey DA Rees A Tiltman A Vercruysse L. Placental Bed Spiral Arteries in the Hypertensive Disorders of Pregnancy. Br J Obstet Gynaecol (1991) 98(7):648–55. doi: 10.1111/j.1471-0528.1991.tb13450.x 1883787

[15] Meekins JW Pijnenborg R Hanssens M McFadyen IR van Asshe A. A Study of Placental Bed Spiral Arteries and Trophoblast Invasion in Normal and Severe Pre-Eclamptic Pregnancies. Br J Obstet Gynaecol (1994) 101(8):669–74. doi: 10.1111/j.1471-0528.1994.tb13182.x 7947500

[16] Jain K Kavi V Raghuveer CV Sinha R. Placental Pathology in Pregnancy-Induced Hypertension (PIH) With or Without Intrauterine Growth Retardation. Indian J Pathol Microbiol (2007) 50(3):533–7. 17883125

[17] Sousa FL Sass N Camano L Stavale JN Mesquita MR Souza EV. [Morphology of the Vascular Placental Bed in Chronic Arterial Hypertension]. Rev Assoc Med Bras (1992) (2008) 54(6):537–42. doi: 10.1590/S0104-42302008000600019 19197532

[18] Milosevic-Stevanovic J Krstic M Radovic-Janosevic D Stefanovic M Antic V Djordjevic I. Preeclampsia With and Without Intrauterine Growth Restriction-Two Pathogenetically Different Entities? Hypertens Pregnancy (2016) 35(4):573–82. doi: 10.1080/10641955.2016.1212872 27624400

[19] Tateishi A Ohira S Yamamoto Y Kanno H. Histopathological Findings of Pregnancy-Induced Hypertension: Histopathology of Early-Onset Type Reflects Two-Stage Disorder Theory. Virchows Arch (2018) 472(4):635–42. doi: 10.1007/s00428-018-2315-3 29426962

[20] Moe K Alnaes-Katjavivi P Storvold GL Sugulle M Johnsen GM Redman CWG. Classical Cardiovascular Risk Markers in Pregnancy and Associations to Uteroplacental Acute Atherosis. Hypertension (2018) 72(3):695–702. doi: 10.1161/HYPERTENSIONAHA.118.10964 30354752

[21] Rieber-Mohn AB Sugulle M Wallukat G Alnaes-Katjavivi P Leite Storvold G Bolstad N. Auto-Antibodies Against the Angiotensin II Type I Receptor in Women With Uteroplacental Acute Atherosis and Preeclampsia at Delivery and Several Years Postpartum. J Reprod Immunol (2018) 128:23–9. doi: 10.1016/j.jri.2018.05.008 29843114

[22] Alnaes-Katjavivi P Lyall F Roald B Redman CW Staff AC. Acute Atherosis in Vacuum Suction Biopsies of Decidua Basalis: An Evidence Based Research Definition. Placenta (2016) 37:26–33. doi: 10.1016/j.placenta.2015.10.020 26608629

[23] Harsem NK Roald B Braekke K Staff AC. Acute Atherosis in Decidual Tissue: Not Associated With Systemic Oxidative Stress in Preeclampsia. Placenta (2007) 28(8-9):958–64. doi: 10.1016/j.placenta.2006.11.005 17218009

[24] Johnsen GM Fjeldstad HES Drabbels JJM Haasnoot GW Eikmans M Storvold GL. A Possible Role for HLA-G in Development of Uteroplacental Acute Atherosis in Preeclampsia. J Reprod Immunol (2021) 144:103284. doi: 10.1016/j.jri.2021.103284 33578175

[25] Johnsen GM Storvold GL Drabbels JJM Haasnoot GW Eikmans M Spruyt-Gerritse MJ. The Combination of Maternal KIR-B and Fetal HLA-C2 is Associated With Decidua Basalis Acute Atherosis in Pregnancies With Preeclampsia. J Reprod Immunol (2018) 129:23–9. doi: 10.1016/j.jri.2018.07.005 30055414

[26] Staff AC Ranheim T Khoury J Henriksen T. Increased Contents of Phospholipids, Cholesterol, and Lipid Peroxides in Decidua Basalis in Women With Preeclampsia. Am J Obstet Gynecol (1999) 180(3 Pt 1):587–92. doi: 10.1016/S0002-9378(99)70259-0 10076133

[27] Brosens I Dixon HG. The Anatomy of the Maternal Side of the Placenta. J Obstet Gynaecol Br Commonw (1966) 73(3):357–63. doi: 10.1111/j.1471-0528.1966.tb05175.x 5947429

[28] Brosens I Renaer M. On the Pathogenesis of Placental Infarcts in Pre-Eclampsia. J Obstet Gynaecol Br Commonw (1972) 79(9):794–9. doi: 10.1111/j.1471-0528.1972.tb12922.x 4651288

[29] Lyall F Robson SC Bulmer JN. Spiral Artery Remodeling and Trophoblast Invasion in Preeclampsia and Fetal Growth Restriction: Relationship to Clinical Outcome. Hypertension (2013) 62(6):1046–54. doi: 10.1161/HYPERTENSIONAHA.113.01892 24060885

[30] Kim YM Chaemsaithong P Romero R Shaman M Kim CJ Kim JS. The Frequency of Acute Atherosis in Normal Pregnancy and Preterm Labor, Preeclampsia, Small-for-Gestational Age, Fetal Death and Midtrimester Spontaneous Abortion. J Matern Fetal Neonatal Med (2015) 28(17):2001–9. doi: 10.3109/14767058.2014.976198 PMC4427552 25308204

[31] Staff AC Halvorsen B Ranheim T Henriksen T. Elevated Level of Free 8-Iso-Prostaglandin F2alpha in the Decidua Basalis of Women With Preeclampsia. Am J Obstet Gynecol (1999) 181(5 Pt 1):1211–5. doi: 10.1016/S0002-9378(99)70110-9 10561647

[32] Burton GJ Sebire NJ Myatt L Tannetta D Wang YL Sadovsky Y. Optimising Sample Collection for Placental Research. Placenta (2014) 35(1):9–22. doi: 10.1016/j.placenta.2013.11.005 24290528

[33] Alnaes-Katjavivi P Roald B Staff AC. Uteroplacental Acute Atherosis in Preeclamptic Pregnancies: Rates and Clinical Outcomes Differ by Tissue Collection Methods. Pregnancy Hypertens (2020) 19:11–7. doi: 10.1016/j.preghy.2019.11.007 31864207

[34] Harsem NK Staff AC He L Roald B. The Decidual Suction Method: A New Way of Collecting Decidual Tissue for Functional and Morphological Studies. Acta Obstet Gynecol Scand (2004) 83(8):724–30. doi: 10.1111/j.0001-6349.2004.00395.x 15255844

[35] Fosheim IK Alnaes-Katjavivi P Redman C Roald B Staff AC Storvold GL. Acute Atherosis of Decidua Basalis; Characterization of Spiral Arteries, Endothelial Status and Activation. Placenta (2019) 82:10–6. doi: 10.1016/j.placenta.2019.04.006 31174621

[36] Jacobsen DP Lekva T Moe K Fjeldstad HES Johnsen GM Sugulle M. Pregnancy and Postpartum Levels of Circulating Maternal sHLA-G in Preeclampsia. J Reprod Immunol (2021) 143:103249. doi: 10.1016/j.jri.2020.103249 33254097

[37] Johnsen GM Storvold GL Alnaes-Katjavivi PH Roald B Golic M Dechend R. Lymphocyte Characterization of Decidua Basalis Spiral Arteries With Acute Atherosis in Preeclamptic and Normotensive Pregnancies. J Reprod Immunol (2019) 132:42–8. doi: 10.1016/j.jri.2019.03.003 30928772

[38] Kvehaugen AS Melien O Holmen OL Laivuori H Oian P Andersgaard AB. Single Nucleotide Polymorphisms in G Protein Signaling Pathway Genes in Preeclampsia. Hypertension (2013) 61(3):655–61. doi: 10.1161/HYPERTENSIONAHA.111.00331 23339167

[39] Lekva T Sugulle M Moe K Redman C Dechend R Staff AC. Multiplex Analysis of Circulating Maternal Cardiovascular Biomarkers Comparing Preeclampsia Subtypes. Hypertension (2020) 75(6):1513–22. doi: 10.1161/HYPERTENSIONAHA.119.14580 32336238

[40] Staff AC Ranheim T Halvorsen B. Augmented PLA2 Activity in Pre-Eclamptic Decidual Tissue–a Key Player in the Pathophysiology of ‘Acute Atherosis’ in Pre-Eclampsia? Placenta (2003) 24(10):965–73. doi: 10.1016/S0143-4004(03)00175-9 14580379

[41] Fosheim IK Johnsen GM Alnaes-Katjavivi P Turowski G Sugulle M Staff AC. Decidua Basalis and Acute Atherosis: Expression of Atherosclerotic Foam Cell Associated Proteins. Placenta (2021) 107:1–7. doi: 10.1016/j.placenta.2021.03.001 33725567

[42] De Wolf F Brosens I Robertson WB. Ultrastructure of Uteroplacental Arteries. Contrib Gynecol Obstet (1982) 9:86–99. doi: 10.1159/000406847 6754251

[43] Khong TY Pearce JM Robertson WB. Acute Atherosis in Preeclampsia: Maternal Determinants and Fetal Outcome in the Presence of the Lesion. Am J Obstet Gynecol (1987) 157(2):360–3. doi: 10.1016/S0002-9378(87)80172-2 3618685

[44] Coffey CG. Cellular Bases for the Lipid-Related Aspects of Preeclampsia. Med Hypotheses (2003) 60(5):716–9. doi: 10.1016/S0306-9877(03)00044-6 12710909

[45] Yanagisawa M Kurihara H Kimura S Tomobe Y Kobayashi M Mitsui Y. A Novel Potent Vasoconstrictor Peptide Produced by Vascular Endothelial Cells. Nature (1988) 332(6163):411–5. doi: 10.1038/332411a0 2451132

[46] Macarthur H Warner TD Wood EG Corder R Vane JR. Endothelin-1 Release From Endothelial Cells in Culture is Elevated Both Acutely and Chronically by Short Periods of Mechanical Stretch. Biochem Biophys Res Commun (1994) 200(1):395–400. doi: 10.1006/bbrc.1994.1462 8166711

[47] Rakugi H Tabuchi Y Nakamaru M Nagano M Higashimori K Mikami H. Evidence for Endothelin-1 Release From Resistance Vessels of Rats in Response to Hypoxia. Biochem Biophys Res Commun (1990) 169(3):973–7. doi: 10.1016/0006-291X(90)91989-6 2194458

[48] Clark BA Halvorson L Sachs B Epstein FH. Plasma Endothelin Levels in Preeclampsia: Elevation and Correlation With Uric Acid Levels and Renal Impairment. Am J Obstet Gynecol (1992) 166(3):962–8. doi: 10.1016/0002-9378(92)91372-H 1532292

[49] Lu YP Hasan AA Zeng S Hocher B. Plasma ET-1 Concentrations Are Elevated in Pregnant Women With Hypertension -Meta-Analysis of Clinical Studies. Kidney Blood Press Res (2017) 42(4):654–63. doi: 10.1159/000482004 29212079

[50] Han SG Ko S Lee WK Jung ST Yu YG. Determination of the Endothelin-1 Recognition Sites of Endothelin Receptor Type A by the Directed-Degeneration Method. Sci Rep (2017) 7(1):7577. doi: 10.1038/s41598-017-08096-6 28790412 PMC5548930

[51] Lin CY Lee TS Chen CC Chang CA Lin YJ Hsu YP. Endothelin-1 Exacerbates Lipid Accumulation by Increasing the Protein Degradation of the ATP-Binding Cassette Transporter G1 in Macrophages. J Cell Physiol (2011) 226(8):2198–205. doi: 10.1002/jcp.22556 21520072

[52] Stryer L Bourne HR. G Proteins: A Family of Signal Transducers. Annu Rev Cell Biol (1986) 2:391–419. doi: 10.1146/annurev.cb.02.110186.002135 3103658

[53] da Silva AR Fraga-Silva RA Stergiopulos N Montecucco F Mach F. Update on the Role of Angiotensin in the Pathophysiology of Coronary Atherothrombosis. Eur J Clin Invest (2015) 45(3):274–87. doi: 10.1111/eci.12401 25586671

[54] Dechend R Muller DN Wallukat G Homuth V Krause M Dudenhausen J. Activating Auto-Antibodies Against the AT1 Receptor in Preeclampsia. Autoimmun Rev (2005) 4(1):61–5. doi: 10.1016/j.autrev.2004.07.002 15652781

[55] Dechend R Viedt C Muller DN Ugele B Brandes RP Wallukat G. AT1 Receptor Agonistic Antibodies From Preeclamptic Patients Stimulate NADPH Oxidase. Circulation (2003) 107(12):1632–9. doi: 10.1161/01.CIR.0000058200.90059.B1 12668498

[56] Herse F Verlohren S Wenzel K Pape J Muller DN Modrow S. Prevalence of Agonistic Autoantibodies Against the Angiotensin II Type 1 Receptor and Soluble Fms-Like Tyrosine Kinase 1 in a Gestational Age-Matched Case Study. Hypertension (2009) 53(2):393–8. doi: 10.1161/HYPERTENSIONAHA.108.124115 19064815

[57] Lin YJ Kwok CF Juan CC Hsu YP Shih KC Chen CC. Angiotensin II Enhances Endothelin-1-Induced Vasoconstriction Through Upregulating Endothelin Type A Receptor. Biochem Biophys Res Commun (2014) 451(2):263–9. doi: 10.1016/j.bbrc.2014.07.119 25088996

[58] Hao J Michalek C Zhang W Zhu M Xu X Mende U. Regulation of Cardiomyocyte Signaling by RGS Proteins: Differential Selectivity Towards G Proteins and Susceptibility to Regulation. J Mol Cell Cardiol (2006) 41(1):51–61. doi: 10.1016/j.yjmcc.2006.04.003 16756988

[59] Nadji P Sommers SC. Lesions of Toxemia in First Trimester Pregnancies. Am J Clin Pathol (1973) 59(3):344–9. doi: 10.1093/ajcp/59.3.344 4687351

[60] Lichtig C Deutch M Brandes J. Vascular Changes of Endometrium in Early Pregnancy. Am J Clin Pathol (1984) 81(6):702–7. doi: 10.1093/ajcp/81.6.702 6233900

[61] Caritis S Sibai B Hauth J Lindheimer M VanDorsten P Klebanoff M. Predictors of Pre-Eclampsia in Women at High Risk. National Institute of Child Health and Human Development Network of Maternal-Fetal Medicine Units. Am J Obstet Gynecol (1998) 179(4):946–51. doi: 10.1016/S0002-9378(98)70194-2 9790376

[62] Luo ZC An N Xu HR Larante A Audibert F Fraser WD. The Effects and Mechanisms of Primiparity on the Risk of Pre-Eclampsia: A Systematic Review. Paediatr Perinat Epidemiol (2007) 21 Suppl 1:36–45. doi: 10.1111/j.1365-3016.2007.00836.x 17593196

[63] Back M Yurdagul A Jr. Tabas I Oorni K Kovanen PT. Inflammation and its Resolution in Atherosclerosis: Mediators and Therapeutic Opportunities. Nat Rev Cardiol (2019) 16(7):389–406. doi: 10.1038/s41569-019-0169-2 30846875 PMC6727648

[64] Brosens I Brosens JJ Muter J Benagiano G. Acute Atherosis and Diffuse Lipid Infiltration of the Placental Bed: A Review of Historical Lipid Studies. Placenta (2020) 97:36–41. doi: 10.1016/j.placenta.2020.06.012 32792060

[65] Stary HC Chandler AB Glagov S Guyton JR Insull W Jr. Rosenfeld ME. A Definition of Initial, Fatty Streak, and Intermediate Lesions of Atherosclerosis. A Report From the Committee on Vascular Lesions of the Council on Arteriosclerosis, American Heart Association. Circulation (1994) 89(5):2462–78. doi: 10.1161/01.cir.89.5.2462 8181179

[66] Rath M Niendorf A Reblin T Dietel M Krebber HJ Beisiegel U. Detection and Quantification of Lipoprotein(a) in the Arterial Wall of 107 Coronary Bypass Patients. Arteriosclerosis (1989) 9(5):579–92. doi: 10.1161/01.ATV.9.5.579 2528948

[67] Meekins JW Pijnenborg R Hanssens M van Assche A McFadyen IR. Immunohistochemical Detection of Lipoprotein(a) in the Wall of Placental Bed Spiral Arteries in Normal and Severe Preeclamptic Pregnancies. Placenta (1994) 15(5):511–24. doi: 10.1016/S0143-4004(05)80420-5 7997451

[68] Haukkamaa L Moilanen L Kattainen A Luoto R Kahonen M Leinonen M. Pre-Eclampsia is a Risk Factor of Carotid Artery Atherosclerosis. Cerebrovasc Dis (2009) 27(6):599–607. doi: 10.1159/000216834 19407443

[69] Staff AC Johnsen GM Dechend R Redman CWG. Preeclampsia and Uteroplacental Acute Atherosis: Immune and Inflammatory Factors. J Reprod Immunol (2014) 101-102:120–6. doi: 10.1016/j.jri.2013.09.001 24119981

[70] Okuyama K Yaginuma G Takahashi T Sasaki H Mori S. The Development of Vasa Vasorum of the Human Aorta in Various Conditions. A Morphometric Study. Arch Pathol Lab Med (1988) 112(7):721–5. 3382327

[71] Subbotin VM. Neovascularization of Coronary Tunica Intima (DIT) is the Cause of Coronary Atherosclerosis. Lipoproteins Invade Coronary Intima *via* Neovascularization From Adventitial Vasa Vasorum, But Not From the Arterial Lumen: A Hypothesis. Theor Biol Med Model (2012) 9:11. doi: 10.1186/1742-4682-9-11 22490844 PMC3492120

[72] Subbotin VM. Excessive Intimal Hyperplasia in Human Coronary Arteries Before Intimal Lipid Depositions is the Initiation of Coronary Atherosclerosis and Constitutes a Therapeutic Target. Drug Discovery Today (2016) 21(10):1578–95. doi: 10.1016/j.drudis.2016.05.017 27265770

[73] Arcidiacono MV Rubinat E Borras M Betriu A Trujillano J Vidal T. Left Carotid Adventitial Vasa Vasorum Signal Correlates Directly With Age and With Left Carotid Intima-Media Thickness in Individuals Without Atheromatous Risk Factors. Cardiovasc Ultrasound (2015) 13:20. doi: 10.1186/s12947-015-0014-7 25889409 PMC4404263

[74] Pirillo A Norata GD Catapano AL. LOX-1, OxLDL, and Atherosclerosis. Mediators Inflammation (2013) 2013:152786. doi: 10.1155/2013/152786 PMC3723318 23935243

[75] Davies MJ Gordon JL Gearing AJ Pigott R Woolf N Katz D. The Expression of the Adhesion Molecules ICAM-1, VCAM-1, PECAM, and E-Selectin in Human Atherosclerosis. J Pathol (1993) 171(3):223–9. doi: 10.1002/path.1711710311 7506307

[76] Labarrere CA DiCarlo HL Bammerlin E Hardin JW Kim YM Chaemsaithong P. Failure of Physiologic Transformation of Spiral Arteries, Endothelial and Trophoblast Cell Activation, and Acute Atherosis in the Basal Plate of the Placenta. Am J Obstet Gynecol (2017) 216(3):287 e1– e16. doi: 10.1016/j.ajog.2016.12.029 PMC5881902 28034657

[77] Robertson WB Brosens I Dixon G. Uteroplacental Vascular Pathology. Eur J Obstet Gynecol Reprod Biol (1975) 5(1-2):47–65. doi: 10.1016/0028-2243(75)90130-6 1053577

[78] Drozdz D Latka M Drozdz T Sztefko K Kwinta P. Thrombomodulin as a New Marker of Endothelial Dysfunction in Chronic Kidney Disease in Children. Oxid Med Cell Longev (2018) 2018:1619293. doi: 10.1155/2018/1619293 29682152 PMC5851028

[79] Remkova A Kovacova E Prikazska M Kratochvil’ova H. Thrombomodulin as a Marker of Endothelium Damage in Some Clinical Conditions. Eur J Intern Med (2000) 11(2):79–84. doi: 10.1016/S0953-6205(00)00066-2 10745150

[80] Staff AC Fjeldstad HE Fosheim IK Moe K Turowski G Johnsen GM. Failure of Physiological Transformation and Spiral Artery Atherosis: Their Roles in Preeclampsia. Am J Obstet Gynecol (2020) 225:349. doi: 10.1016/j.ajog.2020.09.026 32971013

[81] Hustin J Foidart JM Lambotte R. Maternal Vascular Lesions in Pre-Eclampsia and Intrauterine Growth Retardation: Light Microscopy and Immunofluorescence. Placenta (1983) 4 Spec No:489–98. 6369298

[82] Labarrere C Alonso J Manni J Domenichini E Althabe O. Immunohistochemical Findings in Acute Atherosis Associated With Intrauterine Growth Retardation. Am J Reprod Immunol Microbiol (1985) 7(4):149–55. doi: 10.1111/j.1600-0897.1985.tb00344.x 3893171

[83] Labarrere CA Catoggio LJ Mullen EG Althabe OH. Placental Lesions in Maternal Autoimmune Diseases. Am J Reprod Immunol Microbiol (1986) 12(3):78–86. doi: 10.1111/j.1600-0897.1986.tb00068.x 3812855

[84] Yin L Han J Ma H. [Uterine and Placental Vascular Lesions in Pregnancy Induced Hypertension and its Relationship to Pregnancy Outcome]. Zhonghua Fu Chan Ke Za Zhi (1998) 33(8):459–61. 10806741

[85] Hering L Herse F Verlohren S Park JK Wellner M Qadri F. Trophoblasts Reduce the Vascular Smooth Muscle Cell Proatherogenic Response. Hypertension (2008) 51(2):554–9. doi: 10.1161/HYPERTENSIONAHA.107.102905 18195163

[86] Abramowsky CR Vegas ME Swinehart G Gyves MT. Decidual Vasculopathy of the Placenta in Lupus Erythematosus. N Engl J Med (1980) 303(12):668–72. doi: 10.1056/NEJM198009183031204 6995842

[87] Khong TY. Immunohistologic Study of the Leukocytic Infiltrate in Maternal Uterine Tissues in Normal and Preeclamptic Pregnancies at Term. Am J Reprod Immunol Microbiol (1987) 15(1):1–8. doi: 10.1111/j.1600-0897.1987.tb00141.x 2892429

[88] Gill N Leng Y Romero R Xu Y Panaitescu B Miller D. The Immunophenotype of Decidual Macrophages in Acute Atherosis. Am J Reprod Immunol (2019) 81(4):e13098. doi: 10.1111/aji.13098 30734977 PMC6556389

[89] Jaiswal N Puri M Agarwal K Singh S Yadav R Tiwary N. COVID-19 as an Independent Risk Factor for Subclinical Placental Dysfunction. Eur J Obstet Gynecol Reprod Biol (2021) 259:7–11. doi: 10.1016/j.ejogrb.2021.01.049 33556768 PMC7845516

[90] Watts RA Scott DG. Recent Developments in the Classification and Assessment of Vasculitis. Best Pract Res Clin Rheumatol (2009) 23(3):429–43. doi: 10.1016/j.berh.2008.12.004 19508949

[91] Jennette JC Falk RJ. Small-Vessel Vasculitis. N Engl J Med (1997) 337(21):1512–23. doi: 10.1056/NEJM199711203372106 9366584

[92] Staff AC. The Two-Stage Placental Model of Preeclampsia: An Update. J Reprod Immunol (2019) 134-135:1–10. doi: 10.1016/j.jri.2019.07.004 31301487

[93] Redman CWG Staff AC Roberts JM. Syncytiotrophoblast Stress in Preeclampsia: The Convergence Point for Multiple Pathways. Am J Obstet Gynecol (2020). doi: 10.1016/j.ajog.2020.09.047 33546842

[94] Kraus TA Engel SM Sperling RS Kellerman L Lo Y Wallenstein S. Characterizing the Pregnancy Immune Phenotype: Results of the Viral Immunity and Pregnancy (VIP) Study. J Clin Immunol (2012) 32(2):300–11. doi: 10.1007/s10875-011-9627-2 PMC7086597 22198680

[95] Aghaeepour N Ganio EA McIlwain D Tsai AS Tingle M Van Gassen S. An Immune Clock of Human Pregnancy. Sci Immunol (2017) 2(15). doi: 10.1126/sciimmunol.aan2946 PMC5701281 28864494

[96] Apps R Murphy SP Fernando R Gardner L Ahad T Moffett A. Human Leucocyte Antigen (HLA) Expression of Primary Trophoblast Cells and Placental Cell Lines, Determined Using Single Antigen Beads to Characterize Allotype Specificities of Anti-HLA Antibodies. Immunology (2009) 127(1):26–39. doi: 10.1111/j.1365-2567.2008.03019.x 19368562 PMC2678179

[97] Ellis SA Sargent IL Redman CW McMichael AJ. Evidence for a Novel HLA Antigen Found on Human Extravillous Trophoblast and a Choriocarcinoma Cell Line. Immunology (1986) 59(4):595–601. 3804380 PMC1453327

[98] Shobu T Sageshima N Tokui H Omura M Saito K Nagatsuka Y. The Surface Expression of HLA-F on Decidual Trophoblasts Increases From Mid to Term Gestation. J Reprod Immunol (2006) 72(1-2):18–32. doi: 10.1016/j.jri.2006.02.001 16806485

[99] Trowsdale J Moffett A. NK Receptor Interactions With MHC Class I Molecules in Pregnancy. Semin Immunol (2008) 20(6):317–20. doi: 10.1016/j.smim.2008.06.002 18656382

[100] King A Hiby SE Verma S Burrows T Gardner L Loke YW. Uterine NK Cells and Trophoblast HLA Class I Molecules. Am J Reprod Immunol (1997) 37(6):459–62. doi: 10.1111/j.1600-0897.1997.tb00260.x 9228302

[101] Dal Porto J Johansen TE Catipovic B Parfiit DJ Tuveson D Gether U. A Soluble Divalent Class I Major Histocompatibility Complex Molecule Inhibits Alloreactive T Cells at Nanomolar Concentrations. Proc Natl Acad Sci U.S.A. (1993) 90(14):6671–5. doi: 10.1073/pnas.90.14.6671 PMC46994 8341685

[102] Contini P Ghio M Poggi A Filaci G Indiveri F Ferrone S. -B,-C and -G Molecules Induce Apoptosis in T and NK CD8+ Cells and Inhibit Cytotoxic T Cell Activity Through CD8 Ligation. Eur J Immunol (2003) 33(1):125–34. doi: 10.1002/immu.200390015 12594841

[103] Spaggiari GM Contini P Carosio R Arvigo M Ghio M Oddone D. Soluble HLA Class I Molecules Induce Natural Killer Cell Apoptosis Through the Engagement of CD8: Evidence for a Negative Regulation Exerted by Members of the Inhibitory Receptor Superfamily. Blood (2002) 99(5):1706–14. doi: 10.1182/blood.V99.5.1706 11861287

[104] Goldman-Wohl DS Ariel I Greenfield C Hochner-Celnikier D Cross J Fisher S. Lack of Human Leukocyte Antigen-G Expression in Extravillous Trophoblasts is Associated With Pre-Eclampsia. Mol Hum Reprod (2000) 6(1):88–95. doi: 10.1093/molehr/6.1.88 10611266

[105] Tilburgs T Scherjon SA van der Mast BJ Haasnoot GW Versteeg VDV-MM Roelen DL. Fetal-Maternal HLA-C Mismatch is Associated With Decidual T Cell Activation and Induction of Functional T Regulatory Cells. J Reprod Immunol (2009) 82(2):148–57. doi: 10.1016/j.jri.2009.05.003 19631389

[106] Hiby SE Walker JJ O’Shaughnessy KM Redman CW Carrington M Trowsdale J. Combinations of Maternal KIR and Fetal HLA-C Genes Influence the Risk of Preeclampsia and Reproductive Success. J Exp Med (2004) 200(8):957–65. doi: 10.1084/jem.20041214 PMC2211839 15477349

[107] Hviid TV. HLA-G in Human Reproduction: Aspects of Genetics, Function and Pregnancy Complications. Hum Reprod Update (2006) 12(3):209–32. doi: 10.1093/humupd/dmi048 16280356

[108] Colbern GT Chiang MH Main EK. Expression of the Nonclassic Histocompatibility Antigen HLA-G by Preeclamptic Placenta. Am J Obstet Gynecol (1994) 170(5 Pt 1):1244–50. doi: 10.1016/S0002-9378(94)70134-2 8178845

[109] Yie SM Li LH Li YM Librach C. HLA-G Protein Concentrations in Maternal Serum and Placental Tissue are Decreased in Preeclampsia. Am J Obstet Gynecol (2004) 191(2):525–9. doi: 10.1016/j.ajog.2004.01.033 15343231

[110] Beneventi F Locatelli E De Amici M Martinetti M Spinillo A. Soluble HLA-G Concentrations in Obese Women During Pregnancy and in Cord Blood. J Reprod Immunol (2017) 119:31–7. doi: 10.1016/j.jri.2016.11.005 27984763

[111] Burton GJ Woods AW Jauniaux E Kingdom JC. Rheological and Physiological Consequences of Conversion of the Maternal Spiral Arteries for Uteroplacental Blood Flow During Human Pregnancy. Placenta (2009) 30(6):473–82. doi: 10.1016/j.placenta.2009.02.009 PMC2697319 19375795

[112] Jauniaux E Watson AL Hempstock J Bao YP Skepper JN Burton GJ. Onset of Maternal Arterial Blood Flow and Placental Oxidative Stress. A Possible Factor in Human Early Pregnancy Failure. Am J Pathol (2000) 157(6):2111–22. doi: 10.1016/S0002-9440(10)64849-3 PMC1885754 11106583

[113] Assali NS Douglass RA Jr. Baird WW Nicholson DB Suyemoto R. Measurement of Uterine Blood Flow and Uterine Metabolism. IV. Results in Normal Pregnancy. Am J Obstet Gynecol (1953) 66(2):248–53. doi: 10.1016/0002-9378(53)90560-2 13065329

[114] Hung TH Burton GJ. Hypoxia and Reoxygenation: A Possible Mechanism for Placental Oxidative Stress in Preeclampsia. Taiwan J Obstet Gynecol (2006) 45(3):189–200. doi: 10.1016/S1028-4559(09)60224-2 17175463

[115] Redman CW Sargent IL Staff AC. IFPA Senior Award Lecture: Making Sense of Pre-Eclampsia - Two Placental Causes of Preeclampsia? Placenta (2014) 35 Suppl:S20–5. doi: 10.1016/j.placenta.2013.12.008 24477207

[116] Xu C Bailly-Maitre B Reed JC. Endoplasmic Reticulum Stress: Cell Life and Death Decisions. J Clin Invest (2005) 115(10):2656–64. doi: 10.1172/JCI26373 PMC1236697 16200199

[117] Burton GJ Yung HW Cindrova-Davies T Charnock-Jones DS. Placental Endoplasmic Reticulum Stress and Oxidative Stress in the Pathophysiology of Unexplained Intrauterine Growth Restriction and Early Onset Preeclampsia. Placenta (2009) 30 Suppl A:S43–8. doi: 10.1016/j.placenta.2008.11.003 PMC2684656 19081132

[118] Cindrova-Davies T Spasic-Boskovic O Jauniaux E Charnock-Jones DS Burton GJ. Nuclear Factor-Kappa B, P38, and Stress-Activated Protein Kinase Mitogen-Activated Protein Kinase Signaling Pathways Regulate Proinflammatory Cytokines and Apoptosis in Human Placental Explants in Response to Oxidative Stress: Effects of Antioxidant Vitamins. Am J Pathol (2007) 170(5):1511–20. doi: 10.2353/ajpath.2007.061035 PMC1854947 17456758

[119] Hung TH Charnock-Jones DS Skepper JN Burton GJ. Secretion of Tumor Necrosis Factor-Alpha From Human Placental Tissues Induced by Hypoxia-Reoxygenation Causes Endothelial Cell Activation *In Vitro*: A Potential Mediator of the Inflammatory Response in Preeclampsia. Am J Pathol (2004) 164(3):1049–61. doi: 10.1016/S0002-9440(10)63192-6 PMC1614718 14982858

[120] Hubel CA. Oxidative Stress in the Pathogenesis of Preeclampsia. Proc Soc Exp Biol Med (1999) 222(3):222–35. doi: 10.1046/j.1525-1373.1999.d01-139.x 10601881

[121] Chiu JJ Chien S. Effects of Disturbed Flow on Vascular Endothelium: Pathophysiological Basis and Clinical Perspectives. Physiol Rev (2011) 91(1):327–87. doi: 10.1152/physrev.00047.2009 PMC3844671 21248169

[122] VanderLaan PA Reardon CA Getz GS. Site Specificity of Atherosclerosis: Site-Selective Responses to Atherosclerotic Modulators. Arterioscler Thromb Vasc Biol (2004) 24(1):12–22. doi: 10.1161/01.ATV.0000105054.43931.f0 14604830

[123] Cornhill JF Herderick EE Stary HC. Topography of Human Aortic Sudanophilic Lesions. Monogr Atheroscler (1990) 15:13–9. 2296239

[124] Chen KD Li YS Kim M Li S Yuan S Chien S. Mechanotransduction in Response to Shear Stress. Roles of Receptor Tyrosine Kinases, Integrins, and Shc. J Biol Chem (1999) 274(26):18393–400. doi: 10.1074/jbc.274.26.18393 10373445

[125] Chien S. Molecular and Mechanical Bases of Focal Lipid Accumulation in Arterial Wall. Prog Biophys Mol Biol (2003) 83(2):131–51. doi: 10.1016/S0079-6107(03)00053-1 12865076

[126] Namiki M Kawashima S Yamashita T Ozaki M Hirase T Ishida T. Local Overexpression of Monocyte Chemoattractant Protein-1 at Vessel Wall Induces Infiltration of Macrophages and Formation of Atherosclerotic Lesion: Synergism With Hypercholesterolemia. Arterioscler Thromb Vasc Biol (2002) 22(1):115–20. doi: 10.1161/hq0102.102278 11788470

[127] Kauma S Takacs P Scordalakes C Walsh S Green K Peng T. Increased Endothelial Monocyte Chemoattractant Protein-1 and Interleukin-8 in Preeclampsia. Obstet Gynecol (2002) 100(4):706–14. doi: 10.1016/s0029-7844(02)02169-5 12383538

[128] Katabuchi H Yih S Ohba T Matsui K Takahashi K Takeya M. Characterization of Macrophages in the Decidual Atherotic Spiral Artery With Special Reference to the Cytology of Foam Cells. Med Electron Microsc (2003) 36(4):253–62. doi: 10.1007/s00795-003-0223-2 16228658

[129] Lorentzen B Henriksen T. Plasma Lipids and Vascular Dysfunction in Preeclampsia. Semin Reprod Endocrinol (1998) 16(1):33–9. doi: 10.1055/s-2007-1016250 9654605

[130] Staff AC Dechend R Pijnenborg R. Learning From the Placenta: Acute Atherosis and Vascular Remodeling in Preeclampsia-Novel Aspects for Atherosclerosis and Future Cardiovascular Health. Hypertension (2010) 56(6):1026–34. doi: 10.1161/HYPERTENSIONAHA.110.157743 20956732

[131] Hirschhorn T Stockwell BR. The Development of the Concept of Ferroptosis. Free Radic Biol Med (2019) 133:130–43. doi: 10.1016/j.freeradbiomed.2018.09.043 PMC6368883 30268886

[132] Dixon SJ Lemberg KM Lamprecht MR Skouta R Zaitsev EM Gleason CE. Ferroptosis: An Iron-Dependent Form of Nonapoptotic Cell Death. Cell (2012) 149(5):1060–72. doi: 10.1016/j.cell.2012.03.042 PMC3367386 22632970

[133] Stockwell BR Friedmann Angeli JP Bayir H Bush AI Conrad M Dixon SJ. Ferroptosis: A Regulated Cell Death Nexus Linking Metabolism, Redox Biology, and Disease. Cell (2017) 171(2):273–85. doi: 10.1016/j.cell.2017.09.021 PMC5685180 28985560

[134] Li Y Feng D Wang Z Zhao Y Sun R Tian D. Ischemia-Induced ACSL4 Activation Contributes to Ferroptosis-Mediated Tissue Injury in Intestinal Ischemia/Reperfusion. Cell Death Differ (2019) 26(11):2284–99. doi: 10.1038/s41418-019-0299-4 PMC6889315 30737476

[135] Yan HF Tuo QZ Yin QZ Lei P. The Pathological Role of Ferroptosis in Ischemia/Reperfusion-Related Injury. Zool Res (2020) 41(3):220–30. doi: 10.24272/j.issn.2095-8137.2020.042 PMC7231469 32314558

[136] Lillo-Moya J Rojas-Sole C Munoz-Salamanca D Panieri E Saso L Rodrigo R. Targeting Ferroptosis Against Ischemia/Reperfusion Cardiac Injury. Antioxid (Basel) (2021) 10(5):667. doi: 10.3390/antiox10050667 PMC8145541 33922912

[137] Altamura S Muckenthaler MU. Iron Toxicity in Diseases of Aging: Alzheimer’s Disease, Parkinson’s Disease and Atherosclerosis. J Alzheimers Dis (2009) 16(4):879–95. doi: 10.3233/JAD-2009-1010 19387120

[138] Chen X Li X Xu X Li L Liang N Zhang L. Ferroptosis and Cardiovascular Disease: Role of Free Radical-Induced Lipid Peroxidation. Free Radic Res (2021) 55:1–11. doi: 10.1080/10715762.2021.1876856 33455488

[139] Bai T Li M Liu Y Qiao Z Wang Z. Inhibition of Ferroptosis Alleviates Atherosclerosis Through Attenuating Lipid Peroxidation and Endothelial Dysfunction in Mouse Aortic Endothelial Cell. Free Radic Biol Med (2020) 160:92–102. doi: 10.1016/j.freeradbiomed.2020.07.026 32768568

[140] Ruparelia N Chai JT Fisher EA Choudhury RP. Inflammatory Processes in Cardiovascular Disease: A Route to Targeted Therapies. Nat Rev Cardiol (2017) 14(5):314. doi: 10.1038/nrcardio.2016.185 28300082

[141] Tsalamandris S Antonopoulos AS Oikonomou E Papamikroulis GA Vogiatzi G Papaioannou S. The Role of Inflammation in Diabetes: Current Concepts and Future Perspectives. Eur Cardiol (2019) 14(1):50–9. doi: 10.15420/ecr.2018.33.1 PMC6523054 31131037

[142] Ellulu MS Patimah I Khaza’ai H Rahmat A Abed Y. Obesity and Inflammation: The Linking Mechanism and the Complications. Arch Med Sci (2017) 13(4):851–63. doi: 10.5114/aoms.2016.58928 PMC5507106 28721154

[143] Watts DH Krohn MA Wener MH Eschenbach DA. C-Reactive Protein in Normal Pregnancy. Obstet Gynecol (1991) 77(2):176–80. doi: 10.1097/00006250-199102000-00002 1988876

[144] Boots C Stephenson MD. Does Obesity Increase the Risk of Miscarriage in Spontaneous Conception: A Systematic Review. Semin Reprod Med (2011) 29(6):507–13. doi: 10.1055/s-0031-1293204 22161463

[145] Nobles CJ Mendola P Mumford SL Naimi AI Yeung EH Kim K. Preconception Blood Pressure Levels and Reproductive Outcomes in a Prospective Cohort of Women Attempting Pregnancy. Hypertension (2018) 71(5):904–10. doi: 10.1161/HYPERTENSIONAHA.117.10705 PMC5897130 29610265

[146] Wright AD Nicholson HO Pollock A Taylor KG Betts S. Spontaneous Abortion and Diabetes Mellitus. Postgrad Med J (1983) 59(691):295–8. doi: 10.1136/pgmj.59.691.295 PMC2417435 6878099

[147] Fox R Kitt J Leeson P Aye CYL Lewandowski AJ. Preeclampsia: Risk Factors, Diagnosis, Management, and the Cardiovascular Impact on the Offspring. J Clin Med (2019) 8(10):1625. doi: 10.3390/jcm8101625 PMC6832549 31590294

[148] Han VX Patel S Jones HF Nielsen TC Mohammad SS Hofer MJ. Maternal Acute and Chronic Inflammation in Pregnancy is Associated With Common Neurodevelopmental Disorders: A Systematic Review. Transl Psychiatry (2021) 11(1):71. doi: 10.1038/s41398-021-01198-w 33479207 PMC7820474

[149] Chen S Zhao S Dalman C Karlsson H Gardner R. Association of Maternal Diabetes With Neurodevelopmental Disorders: Autism Spectrum Disorders, Attention-Deficit/Hyperactivity Disorder and Intellectual Disability. Int J Epidemiol (2021) 50(2):459–74. doi: 10.1093/ije/dyaa212 PMC8128461 33221916

[150] Chen KR Yu T Kang L Lien YJ Kuo PL. Childhood Neurodevelopmental Disorders and Maternal Hypertensive Disorder of Pregnancy. Dev Med Child Neurol (2021) 63(9):1107–13. doi: 10.1111/dmcn.14893 33884610

[151] Zhu Z Tang S Deng X Wang Y. Maternal Systemic Lupus Erythematosus, Rheumatoid Arthritis, and Risk for Autism Spectrum Disorders in Offspring: A Meta-Analysis. J Autism Dev Disord (2020) 50(8):2852–9. doi: 10.1007/s10803-020-04400-y 32034648

[152] Chen SW Zhong XS Jiang LN Zheng XY Xiong YQ Ma SJ. Maternal Autoimmune Diseases and the Risk of Autism Spectrum Disorders in Offspring: A Systematic Review and Meta-Analysis. Behav Brain Res (2016) 296:61–9. doi: 10.1016/j.bbr.2015.08.035 26327239

[153] Chakravarty EF Nelson L Krishnan E. Obstetric Hospitalizations in the United States for Women With Systemic Lupus Erythematosus and Rheumatoid Arthritis. Arthritis Rheum (2006) 54(3):899–907. doi: 10.1002/art.21663 16508972

[154] Kishore S Mittal V Majithia V. Obstetric Outcomes in Women With Rheumatoid Arthritis: Results From Nationwide Inpatient Sample Database 2003-2011(). Semin Arthritis Rheum (2019) 49(2):236–40. doi: 10.1016/j.semarthrit.2019.03.011 30992155

[155] Labarrere C Althabe O. Chronic Villitis of Unknown Etiology and Maternal Arterial Lesions in Preeclamptic Pregnancies. Eur J Obstet Gynecol Reprod Biol (1985) 20(1):1–11. doi: 10.1016/0028-2243(85)90077-2 4029472

[156] Levy RA Avvad E Oliveira J Porto LC. Placental Pathology in Antiphospholipid Syndrome. Lupus (1998) 7 Suppl 2:S81–5. doi: 10.1177/096120339800700218 9814679

[157] Nayar R Lage JM. Placental Changes in a First Trimester Missed Abortion in Maternal Systemic Lupus Erythematosus With Antiphospholipid Syndrome; a Case Report and Review of the Literature. Hum Pathol (1996) 27(2):201–6. doi: 10.1016/S0046-8177(96)90377-9 8617465

[158] Damgaard C Magnussen K Enevold C Nilsson M Tolker-Nielsen T Holmstrup P. Viable Bacteria Associated With Red Blood Cells and Plasma in Freshly Drawn Blood Donations. PloS One (2015) 10(3):e0120826. doi: 10.1371/journal.pone.0120826 25751254 PMC4353618

[159] Potgieter M Bester J Kell DB Pretorius E. The Dormant Blood Microbiome in Chronic, Inflammatory Diseases. FEMS Microbiol Rev (2015) 39(4):567–91. doi: 10.1093/femsre/fuv013 PMC4487407 25940667

[160] Hou D Zhou X Zhong X Settles ML Herring J Wang L. Microbiota of the Seminal Fluid From Healthy and Infertile Men. Fertil Steril (2013) 100(5):1261–9. doi: 10.1016/j.fertnstert.2013.07.1991 PMC3888793 23993888

[161] Aagaard K Ma J Antony KM Ganu R Petrosino J Versalovic J. The Placenta Harbors a Unique Microbiome. Sci Transl Med (2014) 6(237):237ra65. doi: 10.1126/scitranslmed.3008599 PMC4929217 24848255

[162] de Goffau MC Lager S Sovio U Gaccioli F Cook E Peacock SJ. Human Placenta has No Microbiome But can Contain Potential Pathogens. Nature (2019) 572(7769):329–34. doi: 10.1038/s41586-019-1451-5 PMC6697540 31367035

[163] Swank GM Deitch EA. Role of the Gut in Multiple Organ Failure: Bacterial Translocation and Permeability Changes. World J Surg (1996) 20(4):411–7. doi: 10.1007/s002689900065 8662128

[164] Bhanji S Williams B Sheller B Elwood T Mancl L. Transient Bacteremia Induced by Toothbrushing a Comparison of the Sonicare Toothbrush With a Conventional Toothbrush. Pediatr Dent (2002) 24(4):295–9. 12212870

[165] Flores-Mireles AL Walker JN Caparon M Hultgren SJ. Urinary Tract Infections: Epidemiology, Mechanisms of Infection and Treatment Options. Nat Rev Microbiol (2015) 13(5):269–84. doi: 10.1038/nrmicro3432 PMC4457377 25853778

[166] Kell DB Pretorius E. No Effects Without Causes: The Iron Dysregulation and Dormant Microbes Hypothesis for Chronic, Inflammatory Diseases. Biol Rev Camb Philos Soc (2018) 93(3):1518–57. doi: 10.1111/brv.12407 PMC6055827 29575574

[167] Lannergard A Larsson A Friman G Ewald U. Human Serum Amyloid A (SAA) and High Sensitive C-Reactive Protein (hsCRP) in Preterm Newborn Infants With Nosocomial Infections. Acta Paediatr (2008) 97(8):1061–5. doi: 10.1111/j.1651-2227.2008.00814.x 18510717

[168] Kell DB Pretorius E. On the Translocation of Bacteria and Their Lipopolysaccharides Between Blood and Peripheral Locations in Chronic, Inflammatory Diseases: The Central Roles of LPS and LPS-Induced Cell Death. Integr Biol (Camb) (2015) 7(11):1339–77. doi: 10.1039/c5ib00158g 26345428

[169] Kell DB Kenny LC. A Dormant Microbial Component in the Development of Preeclampsia. Front Med (Lausanne) (2016) 3:60. doi: 10.3389/fmed.2016.00060 27965958 PMC5126693

[170] Xie F Hu Y Magee LA Money DM Patrick DM Brunham RM. Chlamydia Pneumoniae Infection in Preeclampsia. Hypertens Pregnancy (2010) 29(4):468–77. doi: 10.3109/10641950903242642 20818953

[171] El-Shourbagy MA El-Refaie TA Sayed KK Wahba KA El-Din AS Fathy MM. Impact of Seroconversion and Antichlamydial Treatment on the Rate of Pre-Eclampsia Among Egyptian Primigravidae. Int J Gynaecol Obstet (2011) 113(2):137–40. doi: 10.1016/j.ijgo.2010.11.014 21334621

[172] Ponzetto A Cardaropoli S Piccoli E Rolfo A Gennero L Kanduc D. Pre-Eclampsia is Associated With Helicobacter Pylori Seropositivity in Italy. J Hypertens (2006) 24(12):2445–9. doi: 10.1097/HJH.0b013e3280109e8c 17082728

[173] Shapiro NI Yano K Okada H Fischer C Howell M Spokes KC. A Prospective, Observational Study of Soluble FLT-1 and Vascular Endothelial Growth Factor in Sepsis. Shock (2008) 29(4):452–7. doi: 10.1097/SHK.0b013e31815072c1 PMC5378494 18598002

[174] Yano K Liaw PC Mullington JM Shih SC Okada H Bodyak N. Vascular Endothelial Growth Factor is an Important Determinant of Sepsis Morbidity and Mortality. J Exp Med (2006) 203(6):1447–58. doi: 10.1084/jem.20060375 PMC2118329 16702604

[175] Todros T Verdiglione P Ogge G Paladini D Vergani P Cardaropoli S. Low Incidence of Hypertensive Disorders of Pregnancy in Women Treated With Spiramycin for Toxoplasma Infection. Br J Clin Pharmacol (2006) 61(3):336–40. doi: 10.1111/j.1365-2125.2005.02572.x PMC1885027 16487228

[176] Sanchez-Rodriguez E Egea-Zorrilla A Plaza-Diaz J Aragon-Vela J Munoz-Quezada S Tercedor-Sanchez L. The Gut Microbiota and Its Implication in the Development of Atherosclerosis and Related Cardiovascular Diseases. Nutrients (2020) 12(3):605. doi: 10.3390/nu12030605 PMC7146472 32110880

[177] Kiechl S Egger G Mayr M Wiedermann CJ Bonora E Oberhollenzer F. Chronic Infections and the Risk of Carotid Atherosclerosis: Prospective Results From a Large Population Study. Circulation (2001) 103(8):1064–70. doi: 10.1161/01.CIR.103.8.1064 11222467

[178] Serrano M Moreno-Navarrete JM Puig J Moreno M Guerra E Ortega F. Serum Lipopolysaccharide-Binding Protein as a Marker of Atherosclerosis. Atherosclerosis (2013) 230(2):223–7. doi: 10.1016/j.atherosclerosis.2013.07.004 24075748

[179] Ostos MA Recalde D Zakin MM Scott-Algara D. Implication of Natural Killer T Cells in Atherosclerosis Development During a LPS-Induced Chronic Inflammation. FEBS Lett (2002) 519(1-3):23–9. doi: 10.1016/S0014-5793(02)02692-3 12023012

[180] Khedoe PP Wong MC Wagenaar GT Plomp JJ van Eck M Havekes LM. The Effect of PPE-Induced Emphysema and Chronic LPS-Induced Pulmonary Inflammation on Atherosclerosis Development in APOE*3-LEIDEN Mice. PloS One (2013) 8(11):e80196. doi: 10.1371/journal.pone.0080196 24303000 PMC3841138

[181] Koren O Spor A Felin J Fak F Stombaugh J Tremaroli V. Human Oral, Gut, and Plaque Microbiota in Patients With Atherosclerosis. Proc Natl Acad Sci U.S.A. (2011) 108 Suppl 1:4592–8. doi: 10.1073/pnas.1011383107 PMC3063583 20937873

[182] Ott SJ El Mokhtari NE Musfeldt M Hellmig S Freitag S Rehman A. Detection of Diverse Bacterial Signatures in Atherosclerotic Lesions of Patients With Coronary Heart Disease. Circulation (2006) 113(7):929–37. doi: 10.1161/CIRCULATIONAHA.105.579979 16490835

[183] von Dadelszen P Magee LA. Could an Infectious Trigger Explain the Differential Maternal Response to the Shared Placental Pathology of Preeclampsia and Normotensive Intrauterine Growth Restriction? Acta Obstet Gynecol Scand (2002) 81(7):642–8. doi: 10.1080/j.1600-0412.2002.810710.x 12190839

[184] Barak S Oettinger-Barak O Machtei EE Sprecher H Ohel G. Evidence of Periopathogenic Microorganisms in Placentas of Women With Preeclampsia. J Periodontol (2007) 78(4):670–6. doi: 10.1902/jop.2007.060362 17397314

[185] Fjeldstad HE Johnsen GM Staff AC. Fetal Microchimerism and Implications for Maternal Health. Obstet Med (2020) 13(3):112–9. doi: 10.1177/1753495X19884484 PMC7543167 33093862

[186] Schroder J Tiilikainen A de la Chapelle A. Fetal Leukocytes in the Maternal Circulation After Delivery. I. Cytological Aspects. Transplantation (1974) 17(4):346–54. 4823382

[187] Kara RJ Bolli P Karakikes I Matsunaga I Tripodi J Tanweer O. Fetal Cells Traffic to Injured Maternal Myocardium and Undergo Cardiac Differentiation. Circ Res (2012) 110(1):82–93. doi: 10.1161/CIRCRESAHA.111.249037 22082491 PMC3365532

[188] Lo YM Zhang J Leung TN Lau TK Chang AM Hjelm NM. Rapid Clearance of Fetal DNA From Maternal Plasma. Am J Hum Genet (1999) 64(1):218–24. doi: 10.1086/302205 PMC1377720 9915961

[189] Bianchi DW Zickwolf GK Weil GJ Sylvester S DeMaria MA. Male Fetal Progenitor Cells Persist in Maternal Blood for as Long as 27 Years Postpartum. Proc Natl Acad Sci U.S.A. (1996) 93(2):705–8. doi: 10.1073/pnas.93.2.705 PMC40117 8570620

[190] Fugazzola L Cirello V Beck-Peccoz P. Microchimerism and Endocrine Disorders. J Clin Endocrinol Metab (2012) 97(5):1452–61. doi: 10.1210/jc.2011-3160 22399520

[191] Adams Waldorf KM Nelson JL. Autoimmune Disease During Pregnancy and the Microchimerism Legacy of Pregnancy. Immunol Invest (2008) 37(5):631–44. doi: 10.1080/08820130802205886 PMC2709983 18716941

[192] Invernizzi P Pasini S Selmi C Gershwin ME Podda M. Female Predominance and X Chromosome Defects in Autoimmune Diseases. J Autoimmun (2009) 33(1):12–6. doi: 10.1016/j.jaut.2009.03.005 19356902

[193] Gammill HS Aydelotte TM Guthrie KA Nkwopara EC Nelson JL. Cellular Fetal Microchimerism in Preeclampsia. Hypertension (2013) 62(6):1062–7. doi: 10.1161/HYPERTENSIONAHA.113.01486 PMC4395136 24101661

[194] Holzgreve W Ghezzi F Di Naro E Ganshirt D Maymon E Hahn S. Disturbed Feto-Maternal Cell Traffic in Preeclampsia. Obstet Gynecol (1998) 91(5 Pt 1):669–72. doi: 10.1016/s0029-7844(98)00068-4 9572208

[195] Al-Mufti R Lees C Albaiges G Hambley H Nicolaides KH. Fetal Cells in Maternal Blood of Pregnancies With Severe Fetal Growth Restriction. Hum Reprod (2000) 15(1):218–21. doi: 10.1093/humrep/15.1.218 10611215

[196] Gammill HS Nelson JL. Naturally Acquired Microchimerism. Int J Dev Biol (2010) 54(2-3):531–43. doi: 10.1387/ijdb.082767hg PMC2887685 19924635

[197] Kamper-Jorgensen M Hjalgrim H Andersen AM Gadi VK Tjonneland A. Male Microchimerism and Survival Among Women. Int J Epidemiol (2014) 43(1):168–73. doi: 10.1093/ije/dyt230 24345850

[198] Hallum S Gerds TA Sehested TSG Jakobsen MA Tjonneland A Kamper-Jorgensen M. Impact of Male-Origin Microchimerism on Cardiovascular Disease in Women: A Prospective Cohort Study. Am J Epidemiol (2021) 190(5):853–63. doi: 10.1093/aje/kwaa250 33184639

[199] Eikmans M van Halteren AG van Besien K van Rood JJ Drabbels JJ Claas FH. Naturally Acquired Microchimerism: Implications for Transplantation Outcome and Novel Methodologies for Detection. Chimerism (2014) 5(2):24–39. doi: 10.4161/chim.28908 24762743 PMC4199805

[200] Starzl TE Demetris AJ Murase N Ildstad S Ricordi C Trucco M. Cell Migration, Chimerism, and Graft Acceptance. Lancet (1992) 339(8809):1579–82. doi: 10.1016/0140-6736(92)91840-5 PMC2950640 1351558

[201] Schlitt HJ Hundrieser J Ringe B Pichlmayr R. Donor-Type Microchimerism Associated With Graft Rejection Eight Years After Liver Transplantation. N Engl J Med (1994) 330(9):646–7. doi: 10.1056/NEJM199403033300919 8302359

[202] Dempster WJ Harrison CV Shackman R. Rejection Processes in Human Homotransplanted Kidneys. Br Med J (1964) 2(5415):969–76. doi: 10.1136/bmj.2.5415.969 PMC1816585 14185685

[203] Demetris AJ Lasky S Van Thiel DH Starzl TE Dekker A. Pathology of Hepatic Transplantation: A Review of 62 Adult Allograft Recipients Immunosuppressed With a Cyclosporine/Steroid Regimen. Am J Pathol (1985) 118(1):151–61. PMC1887859 3881037

[204] Palmer DC Tsai CC Roodman ST Codd JE Miller LW Sarafian JE. Heart Graft Arteriosclerosis. An Ominous Finding on Endomyocardial Biopsy. Transplantation (1985) 39(4):385–8. doi: 10.1097/00007890-198504000-00009 3885488

[205] Labarrere CA. Acute Atherosis. A Histopathological Hallmark of Immune Aggression? Placenta (1988) 9(1):95–108. doi: 10.1016/0143-4004(88)90076-8 3283724

[206] De Wolf F Carreras LO Moerman P Vermylen J Van Assche A Renaer M. Decidual Vasculopathy and Extensive Placental Infarction in a Patient With Repeated Thromboembolic Accidents, Recurrent Fetal Loss, and a Lupus Anticoagulant. Am J Obstet Gynecol (1982) 142(7):829–34. doi: 10.1016/S0002-9378(16)32527-3 6801984

[207] Stevens DU Al-Nasiry S Bulten J Spaanderman ME. Decidual Vasculopathy in Preeclampsia: Lesion Characteristics Relate to Disease Severity and Perinatal Outcome. Placenta (2013) 34(9):805–9. doi: 10.1016/j.placenta.2013.05.008 23827236

[208] Khong TY Mott C. Immunohistologic Demonstration of Endothelial Disruption in Acute Atherosis in Pre-Eclampsia. Eur J Obstet Gynecol Reprod Biol (1993) 51(3):193–7. doi: 10.1016/0028-2243(93)90034-A 8288014

[209] Mistry DA Chandratreya A Lee PYF. A Systematic Review on the Use of Aspirin in the Prevention of Deep Vein Thrombosis in Major Elective Lower Limb Orthopedic Surgery: An Update From the Past 3 Years. Surg J (N Y) (2017) 3(4):e191–e6. doi: 10.1055/s-0037-1615817 PMC5747531 29302621

[210] Ittaman SV VanWormer JJ Rezkalla SH. The Role of Aspirin in the Prevention of Cardiovascular Disease. Clin Med Res (2014) 12(3-4):147–54. doi: 10.3121/cmr.2013.1197 PMC4317158 24573704

[211] Van Doorn R Mukhtarova N Flyke IP Lasarev M Kim K Hennekens CH. Dose of Aspirin to Prevent Preterm Preeclampsia in Women With Moderate or High-Risk Factors: A Systematic Review and Meta-Analysis. PloS One (2021) 16(3):e0247782. doi: 10.1371/journal.pone.0247782 33690642 PMC7943022

[212] Rolnik DL Wright D Poon LC O’Gorman N Syngelaki A de Paco Matallana C. Aspirin Versus Placebo in Pregnancies at High Risk for Preterm Preeclampsia. N Engl J Med (2017) 377(7):613–22. doi: 10.1056/NEJMoa1704559 28657417

[213] Brown MA Magee LA Kenny LC Karumanchi SA McCarthy FP Saito S. The Hypertensive Disorders of Pregnancy: ISSHP Classification, Diagnosis & Management Recommendations for International Practice. Pregnancy Hypertens (2018) 13:291–310. doi: 10.1016/j.preghy.2018.05.004 29803330

[214] Olofsson P Laurini RN Marsal K. A High Uterine Artery Pulsatility Index Reflects a Defective Development of Placental Bed Spiral Arteries in Pregnancies Complicated by Hypertension and Fetal Growth Retardation. Eur J Obstet Gynecol Reprod Biol (1993) 49(3):161–8. doi: 10.1016/0028-2243(93)90265-E 8405630

[215] Kim YM Chaemsaithong P Romero R Shaman M Kim CJ Kim JS. Placental Lesions Associated With Acute Atherosis. J Matern Fetal Neonatal Med (2015) 28(13):1554–62. doi: 10.3109/14767058.2014.960835 PMC4416076 25183023

[216] Ferrazzi E Bulfamante G Mezzopane R Barbera A Ghidini A Pardi G. Uterine Doppler Velocimetry and Placental Hypoxic-Ischemic Lesion in Pregnancies With Fetal Intrauterine Growth Restriction. Placenta (1999) 20(5-6):389–94. doi: 10.1053/plac.1999.0395 10419803

[217] Aardema MW Oosterhof H Timmer A van Rooy I Aarnoudse JG. Uterine Artery Doppler Flow and Uteroplacental Vascular Pathology in Normal Pregnancies and Pregnancies Complicated by Pre-Eclampsia and Small for Gestational Age Fetuses. Placenta (2001) 22(5):405–11. doi: 10.1053/plac.2001.0676 11373150

[218] Lloyd-Davies C Collins SL Burton GJ. Understanding the Uterine Artery Doppler Waveform and its Relationship to Spiral Artery Remodelling. Placenta (2021) 105:78–84. doi: 10.1016/j.placenta.2021.01.004 33556717

[219] Pijnenborg R Vercruysse L Hanssens M. The Uterine Spiral Arteries in Human Pregnancy: Facts and Controversies. Placenta (2006) 27(9-10):939–58. doi: 10.1016/j.placenta.2005.12.006 16490251

[220] Williams D. Pregnancy: A Stress Test for Life. Curr Opin Obstet Gynecol (2003) 15(6):465–71. doi: 10.1097/00001703-200312000-00002 14624211

[221] Sattar N Greer IA. Pregnancy Complications and Maternal Cardiovascular Risk: Opportunities for Intervention and Screening? BMJ (2002) 325(7356):157–60. doi: 10.1136/bmj.325.7356.157 PMC1123678 12130616

[222] Staff AC Redman CW Williams D Leeson P Moe K Thilaganathan B. Pregnancy and Long-Term Maternal Cardiovascular Health: Progress Through Harmonization of Research Cohorts and Biobanks. Hypertension (2016) 67(2):251–60. doi: 10.1161/HYPERTENSIONAHA.115.06357 26667417

[223] Bushnell C McCullough LD Awad IA Chireau MV Fedder WN Furie KL. Guidelines for the Prevention of Stroke in Women: A Statement for Healthcare Professionals From the American Heart Association/American Stroke Association. Stroke (2014) 45(5):1545–88. doi: 10.1161/01.str.0000442009.06663.48 PMC10152977 24503673

[224] Roberts JM Gammill H. Pre-Eclampsia and Cardiovascular Disease in Later Life. Lancet (2005) 366(9490):961–2. doi: 10.1016/S0140-6736(05)67349-7 16168757

[225] Cain MA Salemi JL Tanner JP Kirby RS Salihu HM Louis JM. Pregnancy as a Window to Future Health: Maternal Placental Syndromes and Short-Term Cardiovascular Outcomes. Am J Obstet Gynecol (2016) 215(4):484 e1– e14. doi: 10.1016/j.ajog.2016.05.047 27263996

[226] Irgens HU Reisaeter L Irgens LM Lie RT. Long Term Mortality of Mothers and Fathers After Pre-Eclampsia: Population Based Cohort Study. BMJ (2001) 323(7323):1213–7. doi: 10.1136/bmj.323.7323.1213 PMC59993 11719411

[227] Smith GC Pell JP Walsh D. Pregnancy Complications and Maternal Risk of Ischaemic Heart Disease: A Retrospective Cohort Study of 129,290 Births. Lancet (2001) 357(9273):2002–6. doi: 10.1016/S0140-6736(00)05112-6 11438131

[228] Bellamy L Casas JP Hingorani AD Williams DJ. Pre-Eclampsia and Risk of Cardiovascular Disease and Cancer in Later Life: Systematic Review and Meta-Analysis. BMJ (2007) 335(7627):974. doi: 10.1136/bmj.39335.385301.BE 17975258 PMC2072042

[229] Lykke JA Langhoff-Roos J Sibai BM Funai EF Triche EW Paidas MJ. Hypertensive Pregnancy Disorders and Subsequent Cardiovascular Morbidity and Type 2 Diabetes Mellitus in the Mother. Hypertension (2009) 53(6):944–51. doi: 10.1161/HYPERTENSIONAHA.109.130765 19433776

[230] Lazdam M de la Horra A Diesch J Kenworthy Y Davis E Lewandowski AJ. Unique Blood Pressure Characteristics in Mother and Offspring After Early Onset Preeclampsia. Hypertension (2012) 60(5):1338–45. doi: 10.1161/HYPERTENSIONAHA.112.198366 23045462

[231] Wikstrom AK Haglund B Olovsson M Lindeberg SN. The Risk of Maternal Ischaemic Heart Disease After Gestational Hypertensive Disease. BJOG (2005) 112(11):1486–91. doi: 10.1111/j.1471-0528.2005.00733.x 16225567

[232] Newstead J von Dadelszen P Magee LA. Preeclampsia and Future Cardiovascular Risk. Expert Rev Cardiovasc Ther (2007) 5(2):283–94. doi: 10.1586/14779072.5.2.283 17338672

[233] Sundheimer LW Pisarska MD. Abnormal Placentation Associated With Infertility as a Marker of Overall Health. Semin Reprod Med (2017) 35(3):205–16. doi: 10.1055/s-0037-1603570 PMC7138513 28658703

[234] Parikh NI Cnattingius S Mittleman MA Ludvigsson JF Ingelsson E. Subfertility and Risk of Later Life Maternal Cardiovascular Disease. Hum Reprod (2012) 27(2):568–75. doi: 10.1093/humrep/der400 PMC3283089 22131387

[235] Milic NM Milin-Lazovic J Weissgerber TL Trajkovic G White WM Garovic VD. Preclinical Atherosclerosis at the Time of Pre-Eclamptic Pregnancy and Up to 10 Years Postpartum: Systematic Review and Meta-Analysis. Ultrasound Obstet Gynecol (2017) 49(1):110–5. doi: 10.1002/uog.17367 PMC5310936 27859887

[236] Yuan LJ Xue D Duan YY Cao TS Yang HG Zhou N. Carotid Arterial Intima-Media Thickness and Arterial Stiffness in Pre-Eclampsia: Analysis With a Radiofrequency Ultrasound Technique. Ultrasound Obstet Gynecol (2013) 42(6):644–52. doi: 10.1002/uog.12409 23335074

[237] Brueckmann A Seeliger C Schlembach D Schleussner E. PP048. Carotid Intima-Media-Thickness in the First Trimester as a Predictor of Preeclampsia. Pregnancy Hypertens (2013) 3(2):84. doi: 10.1016/j.preghy.2013.04.075 26105905

[238] Willeit P Tschiderer L Allara E Reuber K Seekircher L Gao L. Carotid Intima-Media Thickness Progression as Surrogate Marker for Cardiovascular Risk: Meta-Analysis of 119 Clinical Trials Involving 100 667 Patients. Circulation (2020) 142(7):621–42. doi: 10.1161/CIRCULATIONAHA.120.046361 PMC7115957 32546049

[239] Blaauw J Souwer ET Coffeng SM Smit AJ van Doormaal JJ Faas MM. Follow Up of Intima-Media Thickness After Severe Early-Onset Preeclampsia. Acta Obstet Gynecol Scand (2014) 93(12):1309–16. doi: 10.1111/aogs.12499 25200856

[240] Sandvik MK Leirgul E Nygard O Ueland PM Berg A Svarstad E. Preeclampsia in Healthy Women and Endothelial Dysfunction 10 Years Later. Am J Obstet Gynecol (2013) 209(6):569 e1– e10. doi: 10.1016/j.ajog.2013.07.024 23899451

[241] Akhter T Larsson M Wikstrom AK Naessen T. Thicknesses of Individual Layers of Artery Wall Indicate Increased Cardiovascular Risk in Severe Pre-Eclampsia. Ultrasound Obstet Gynecol (2014) 43(6):675–80. doi: 10.1002/uog.13289 24375803

[242] Stevens DU Smits MP Bulten J Spaanderman ME van Vugt JM Al-Nasiry S. Prevalence of Hypertensive Disorders in Women After Preeclamptic Pregnancy Associated With Decidual Vasculopathy. Hypertens Pregnancy (2015) 34(3):332–41. doi: 10.3109/10641955.2015.1034803 25954825

[243] Stevens DU Al-Nasiry S Fajta MM Bulten J van Dijk AP van der Vlugt MJ. Cardiovascular and Thrombogenic Risk of Decidual Vasculopathy in Preeclampsia. Am J Obstet Gynecol (2014) 210(6):545 e1–6. doi: 10.1016/j.ajog.2013.12.029 24370690

[244] Robertson WB Brosens I Dixon HG. The Pathological Response of the Vessels of the Placental Bed to Hypertensive Pregnancy. J Pathol Bacteriol (1967) 93(2):581–92. doi: 10.1002/path.1700930219 6054058

[245] Veerbeek JH Brouwers L Koster MP Koenen SV van Vliet EO Nikkels PG. Spiral Artery Remodeling and Maternal Cardiovascular Risk: The Spiral Artery Remodeling (SPAR) Study. J Hypertens (2016) 34(8):1570–7. doi: 10.1097/HJH.0000000000000964 27219485

[246] Kim JY Kim YM. Acute Atherosis of the Uterine Spiral Arteries: Clinicopathologic Implications. J Pathol Transl Med (2015) 49(6):462–71. doi: 10.4132/jptm.2015.10.23 PMC4696535 26530045

[247] Staff AC Dechend R Redman CW. Review: Preeclampsia, Acute Atherosis of the Spiral Arteries and Future Cardiovascular Disease: Two New Hypotheses. Placenta (2013) 34 Suppl:S73–8. doi: 10.1016/j.placenta.2012.11.022 23246096

[248] Parks WT Catov JM. The Placenta as a Window to Maternal Vascular Health. Obstet Gynecol Clin North Am (2020) 47(1):17–28. doi: 10.1016/j.ogc.2019.10.001 32008667

[249] Brosens I Benagiano M Puttemans P D’Elios MM Benagiano G. The Placental Bed Vascular Pathology Revisited: A Risk Indicator for Cardiovascular Disease. J Matern Fetal Neonatal Med (2019) 32(9):1556–64. doi: 10.1080/14767058.2017.1409718 29172831

